# KBPT: knowledge-based prompt tuning for zero-shot relation triplet extraction

**DOI:** 10.7717/peerj-cs.2014

**Published:** 2024-05-24

**Authors:** Qian Guo, Yi Guo, Jin Zhao

**Affiliations:** 1 Department of Computer Science and Engineering, East China University of Science and Technology, Shanghai, China; 2 School of Computer Science, Fudan University, Shanghai, China

**Keywords:** Knowledge-based, Zero-shot relation triplet extraction, Prompt tuning

## Abstract

Knowledge representation is increasingly recognized as an effective method for information extraction. Nevertheless, numerous studies have disregarded its potential applications in the zero-shot setting. In this article, a novel framework, called knowledge-based prompt tuning for zero-shot relation triplet extraction (KBPT), was developed, founded on external ontology knowledge. This framework serves as a catalyst for exploring relation triplet extraction (RTE) methods within low-resource scenarios, warranting further scrutiny. Zero-shot setting RTE aims to extract multiple triplets that consist of head entities, tail entities, and relation labels from an input sentence, where the extracted relation labels are those that do not exist in the training set. To address the data scarcity problem in zero-shot RTE, a technique was introduced to synthesize training samples by prompting language models to generate structured texts. Specifically, this involves integrating language model prompts with structured text methodologies to create a structured prompt template. This template draws upon relation labels and ontology knowledge to generate synthetic training examples. The incorporation of external ontological knowledge enriches the semantic representation within the prompt template, enhancing its effectiveness. Further, a multiple triplets decoding (MTD) algorithm was developed to overcome the challenge of extracting multiple relation triplets from a sentence. To bridge the gap between knowledge and text, a collective training method was established to jointly optimize embedding representations. The proposed model is model-agnostic and can be applied to various PLMs. Exhaustive experiments on four public datasets with zero-shot settings were conducted to demonstrate the effectiveness of the proposed method. Compared to the baseline models, KBPT demonstrated enhancements of up to 14.65% and 24.19% in F1 score on the Wiki-ZSL and TACRED-Revisit datasets, respectively. Moreover, the proposed model achieved better performance compared with the current state-of-the-art (SOTA) model in terms of F1 score, precision-recall (P–R) curves and AUC. The code is available at https://Github.com/Phevos75/KBPT.

## Introduction

Relation extraction (RE) endeavors to discern and extract the relationship existing between provided entity pairs from unstructured resources. Due to its capability of extracting essential information, RE is widely utilized in various downstream tasks of natural language processing (NLP), such as information retrieval (Yao et al., 2023) and knowledge base construction (Morio et al., 2022). At present, supervised learning (Han et al., 2018) based on labeled training data and distant supervision strategy (Ji et al., 2017) have emerged as prominent methods in RE. However, both methods possess drawbacks. Supervised learning is constrained by the quantity of labeled training samples due to the substantial human and financial resources required for manual labeling. Moreover, training samples generated by distant supervision tend to be noisy, further complicating the learning process. To address the aforementioned challenge, few-shot learning (Ye et al., 2020) has emerged as a promising approach. This method requires only a small amount of annotated data to achieve remarkable training performance. The earliest application of few-shot learning originated from computer vision, and many subsequent studies (Lifchitz et al., 2019; Li et al., 2020; Ye et al., 2020) have emerged since then. Research has gradually expanded to other application fields, such as information extraction (Gao et al., 2019; Hui et al., 2020) and natural language understanding (NLU) (Gao et al., 2020). Despite the effectiveness of these approaches with a limited amount of training data, they may still be ineffective in scenarios where no annotated samples are available.

The current approaches to address data scarcity can be categorized into three main categories. Firstly, constructing large-scale corpora through distant supervision (Ji et al., 2017), although this method is limited by a fixed number of relation types and suffers from poor annotation quality (Smirnova & Cudré-Mauroux, 2018). Secondly, defining task objectives to accommodate an unconstrained label space, as seen in open information extraction (OIE) (Angeli, Premkumar & Manning, 2015), which directly extracts relations from open corpora. However, this approach may struggle with effectively identifying meaningful relation patterns and filtering out irrelevant information. The third direction involves leveraging pre-trained language models (PLMs) (Lee & Toutanova, 2018) with task-specific prompt templates, enabling models to generalize to new tasks with minimal or no training samples, such as zero-shot classification (Zhong & Chen, 2020). Nonetheless, constructing prompt templates (Cui et al., 2021) can be challenging and may lack generalization ability. A recent study (Chia et al., 2022) suggested the use of relation labels as prompts for the generation of synthetic training samples. The aim of this method is to uncover semantic knowledge in relation labels to represent unseen relations. However, relying solely on relation labels may result in incomplete relational semantics and hinder the generation of high-quality training samples.

**Figure 1:**
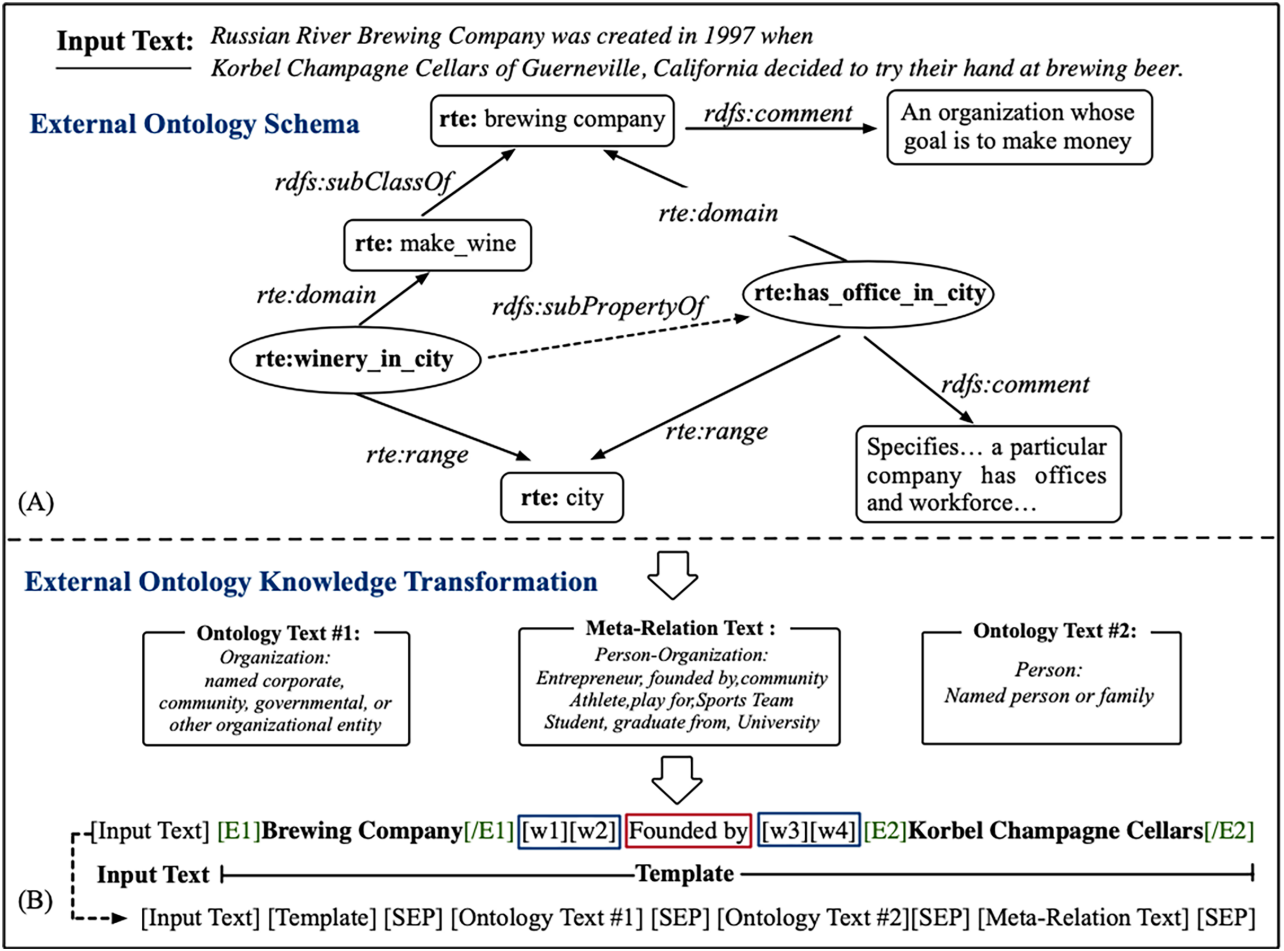
The overall framework of prompt template construction. The formation of the external ontology schema (A) and Ontology Transformation (B). w1;w2 and w3;w4 in the blue squares are the learnable virtual tokens, and it is the relation label in red square, which consisting prompt template together.

The aforementioned methods primarily focus on analyzing relational data characterized by incomplete semantic information. Illustrated in Fig. 1, when the prompt template exclusively features the relational label *Created by* without incorporating detailed conceptual attribute descriptions from the ontology schema (Horrocks, 2008), such as *Organization*, *Person*, *etc*., the resulting synthetic samples are susceptible to semantic incompleteness. Therefore, more expressive prior knowledge is expected to boost the quality of the generated synthetic samples. In the present study, an ontology scheme sourced from an external knowledge base was utilized to enhance prompt information. The ontology schema represents a general pattern of concepts (that is, the types of things) that exist in a specific domain, and the property is used to link the semantic relation between concepts. It can represent a wealth of valuable information, such as concept hierarchy and meta-data (that is, textual definitions, comments, and descriptions of concepts). Several instances of ontology schema are shown in the upper part of Fig. 1, and a more detailed description is shown in ontology knowledge transformation.

While the integration of ontology knowledge enhances understanding of relational semantics to some extent, several significant challenges persist. Primarily, a considerable level of noise is present in the external knowledge base (Ye et al., 2022). Studies (Liu et al., 2020; Zhang et al., 2021a) have shown that not all external knowledge is beneficial for improving task performance, and blind knowledge injection may not be conducive to the model’s performance. As such, it is imperative to curate learnable knowledge that harmonizes the prompt template, thereby enhancing the coding performance of relational triples. Additionally, employing specific markers to encode structured text offers benefits for decoding triples. Moreover, crafting a pragmatic prompt template capable of encoding multiple triples may compromise the generation quality of synthetic samples, given the model’s challenge in processing multiple relations simultaneously. Thus, the focus of the present study was on generating single-triplet samples and investigating how the downstream relation extractor can mitigate this constraint.

In this article, we propose a novel zero-shot relation triplet extraction framework called KBPT. The proposed framework not only enhances the relation semantics with an ontological schema, but also employs an ontology-based generative prompt template to synthesize training samples for unseen relational categories. Specifically, an ontology embedding technique was first proposed for learning meaningful embedding representations of unseen relation categories from the ontological schema. Subsequently, a generative model was employed to produce features and create training samples tailored to the desired relation, effectively transforming the zero-shot setting problem into a supervised learning scenario through empirical methods. This operation is viable and substantiated by research (Keskar et al., 2019), which enables prompting the language model to generate domain-specific structured text. For instance, when presented with the relation label *Created by* as depicted in Fig. 1, it is reasonable to prompt the language model to generate a sentence incorporating the relevant entity. This entity signifies the entity that was uncovered, initially described, or invented, among other possibilities. Therefore, a possible output sentence could be *The county was created in 1818 by the Virginia General*, which contains head entity *Virginia General* and tail entity *The county*, respectively. Therefore, given enough high-quality synthetic training samples, the zero-shot potential is possible by leveraging the semantic information in prompts to transform it into a supervised learning task. In summary, the main contributions of the present study include:

- Knowledge-based prompt tuning (KBPT) was introduced for zero-shot relation triplet extraction, which overcomes the limitation of data sparsity in traditional tasks by expanding the scope of relation triplet extraction to zero-shot scenarios.
- The proposed knowledge-based method not only incorporates prior knowledge from ontological schemas, which enhance semantic representations, but also employs a generative prompt model to synthesize training samples for unseen relational types.
- A multiple triplet decoding (MTD) algorithm was proposed to achieve the goal of extracting multiple relation triplets from a sentence at once.
- Finally, KBPT was evaluated on four public datasets, namely FewRel (https://thunlp.github.io/1/fewrel1.html) (Han et al., 2018), NYT (https://Github.com/davidsbatista/Annotated-Semantic-Relationships-Datasets/blob/master/datasets/DataSet-IJCNLP2011.tar.gz) (Riedel, Yao & McCallum, 2010), TACRED-Revisit (https://paperswithcode.com/dataset/tacred-revisited) (Alt, Gabryszak & Hennig, 2020), and Wiki-ZSL (https://raw.githubusercontent.com/dinobby/ZS-BERT/master/resources/property_list.html) (Chen & Li, 2021). The experimental results illustrate that KBPT attained state-of-the-art performance, producing instances that were both reasonable and diverse. These generated instances can be utilized as training data, thereby establishing a benchmark for subsequent research endeavors.

The overall structure of this article is organized as follows: “Related Works” provides an overview of related works concerning prompt tuning and zero-shot learning. “Knowledge-Based Prompt Tuning Methodology” elaborates on the proposed method, presenting the knowledge-based prompt tuning methodology in detail. “Experiments and Results” encompasses the experiments, which include the presentation of experimental results and their analysis. Finally, “Conclusion” and future work concludes the studies discussed in this article and offers insights into potential avenues for future research.

## Related works

### Zero-shot setting for relation extraction

Zero-shot learning can be broadly categorized into two approaches. The first involves projecting features from seen classes onto the space of unseen classes through mapping, as demonstrated by Kodirov, Xiang & Gong (2017). However, this method is susceptible to domain drift, as highlighted by Tomani et al. (2021). The second approach entails generating synthetic training samples from seen classes to supervise the training of another model, effectively reframing the zero-shot learning challenge as a supervised learning task, as proposed by Qin et al. (2020). Despite utilizing samples from seen classes, the latter method demonstrates strong generalization to unseen classes based on their semantic relations.

The zero-shot setting for relation extraction can be defined as a slot-filling task with the neural reading-comprehension technique (Levy et al., 2017). Nonetheless, such approach can only generate new relation classes well if each relation label template requires manual design. An alternative approach involves conceptualizing the zero-shot relation classification task as textual entailment, leveraging pre-existing textual entailment models (Obamuyide & Vlachos, 2018). However, a drawback of this method is that the space of relation labels is not fixed. Moreover, it is suitable for text classification and does not perform well in zero-shot relation extraction. A recent study (Gong & Eldardiry, 2020) on the zero-shot setting in relation extraction garnered considerable interest. This study utilized prototypical networks to leverage auxiliary information for recognizing new relations. However, the complexity associated with embedding side information hampers the portability of this approach, particularly when applied to the large scale of the zero-shot setting task. The most related research to this study is OntoZSL (Geng et al., 2021). This approach addresses the inadequacies of prior knowledge by incorporating external ontology knowledge. It leverages seen classes to generate synthetic training samples for a range of downstream tasks, including relation extraction. Inspired by this method, knowledge-based prompt tuning (KBPT) was proposed for zero-shot relation triplet extraction. A distinguishing feature is the utilization of the advanced language comprehension capabilities inherent in pre-trained language models. Additionally, the integration of insights from the human wisdom knowledge graph enhances the effectiveness of prompting the language model to generate synthetic training samples. As far as current knowledge suggests, we are the first research team to apply knowledge-based prompt tuning to zero-shot setting relation triplet extraction.

### Prompt tuning for PLMs

Prompt-tuning (Zhang et al., 2021b; Schick & Schütze, 2020) is a new methodology aimed at bridging the divide between PLMs and fine-tuning for downstream tasks. Originally conceptualized as a cloze task, prompt-tuning has evolved into a burgeoning research domain that has garnered significant academic interest in recent times. Prompt tuning with GPT-3 (Brown et al., 2020) has effectively mitigated its limitations and resolved numerous issues associated with fine-tuning across diverse application scenarios in NLP. These scenarios include text classification (Han et al., 2021), relation extraction (Chen et al., 2022b), and other domain-specific tasks (Yeh, Lavergne & Zweigenbaum, 2022). In addition, prompt tuning has also achieved favorable performance in the low-data regime and overcomes deficiencies of GPT-3 with large-scale language models (Gu et al., 2021). The recent work by Chia et al. (2022), termed RelationPrompt, concentrates on leveraging seen relation labels to produce synthetic training samples for unseen relation classes, achieving notable performance. On the other hand, KPT, introduced by Hu et al. (2021), utilizes external knowledge bases to generate label words corresponding to each label. It integrates this external knowledge into the verbalizers to facilitate prompt learning. Although the aforementioned approaches demonstrate relatively good performance, they are constrained by their focus on single semantic knowledge within relation labels or external knowledge. Moreover, they only partially exploit the potential of fused semantic knowledge to complement each other.

Notably, there have been more recent research attempts to inject external knowledge into the prompt template. KnowPrompt (Chen et al., 2022a) leverages latent knowledge embedded within relation labels. It incorporates this knowledge into the prompt template by constructing virtual type words and answer words. This approach significantly enhances the model’s performance. To fully exploit the prior knowledge in the relationship category, Han et al. (2021) proposed prompt tuning with rules (PTR) that leverage logic rules consisting of the sub-prompt for text classification. Knowledgeable prompt-tuning (KPT) (Hu et al., 2021) integrates external knowledge into the verbalizer using a knowledge graph. This process aids in forming the prompt template for text classification tasks. To address the challenge of knowledge gaps, Ye et al. (2022) introduced ontology-enhanced prompt-tuning (OntoPrompt). This method converts structured knowledge into text format using an external knowledge graph. Inspired by these methodologies, we incorporate external ontology knowledge to enrich relation semantics, thereby supplementing incomplete prior knowledge in the prompt template for zero-shot relation triplet extraction.

### Knowledge-enhanced learning

PLMs have exhibited superior performance improvements in various NLP tasks (Brown et al., 2020) by leveraging powerful language representation capabilities. However, direct fine-tuning often results in suboptimal performance, particularly on knowledge-intensive tasks. Thus, the injection of external knowledge plays a certain role in relieving this issue through auxiliary language understanding (Zhang et al., 2022). Recently, Deng et al. (2021) integrated ontology knowledge into the PLMs for event detection, with a considerable performance improvement being achieved. Geng et al. (2021) investigated rich prior knowledge models by incorporating inter-class relationships through ontological knowledge representation. They utilized this approach to generate synthesized training data across various domains in the zero-shot setting. Other approaches obtain knowledge by explicitly injecting entity representation into PLMs. To illustrate, OntoEA (Xiang et al., 2021) adapts the entity alignment approach that relies on ontology schema. It integrates knowledge graphs and ontology knowledge in a joint embedding framework. OntoPrompt (Ye et al., 2022) injects ontological knowledge into a prompt template for few-shot learning. This integration aims to mitigate issues related to knowledge incompleteness and heterogeneity. Yao et al. (2023) proposed a retrieval-augmented approach, which retrieves schema-aware references as prompt (RAP) for data-efficient knowledge graph construction. Several studies used label ontologies, such as the one in OWL (Web Ontology Language) (Chen et al., 2020), to explore interclass relationships. Morio et al. (2022) employed graph-based parsers to capture subjective aspects of structured sentiments for information retrieval. However, this external knowledge also has its limitations. Moreover, while graph-based structures offer valuable insights, they may encounter challenges in comprehending contextual coherence.

KBPT is related to the aforementioned approaches, but there is a considerable difference. First, ontology knowledge sources from the external knowledge base were integrated into PLMs to construct a prompt template, which is different from the aforementioned works. The ontology knowledge used in the present model contains richer semantics, and it is mainly in the form of multi-relational graphs composed of RDF triples, which is easier to model using various successful triple embedding algorithms. This differs from the construction of the ontologies used in previous research (Chen et al., 2020), which heavily depends on manual annotation. Second, a novel ontology transformation strategy was proposed for alleviating the ontology knowledge encoding issue in knowledge-enhanced learning. Third, in addition to the external ontology knowledge, the relation labels and the generated virtual tokens (for example, special tokens in the vocabulary) form the main components of the prompt template, allowing for the semantic knowledge contained in the relational labels to be fully exploited. Finally, collective training is utilized to jointly optimize those parametric representations.

## Knowledge-based prompt tuning methodology

The proposed framework contains two modules: relation data generation(RDG) for synthetic relation samples and relation triplet extraction, which will be trained on the synthetic data and used to predict triplets for unseen relations. In order to fusion ontological prior knowledge, we design structured prompt templates. We further propose a multiple triplet decoding (MTD) algorithm to overcome the challenge of generating relation samples with multiple triplets from a sentence.

### Preliminary work with prompt tuning

#### Zero-shot setting preliminary

Zero-shot setting relation triplet extraction aims to recognize the new relation triplets that do not exist in the training set ${D_s}$. We define ${D_s} = \{(x,y)|x \in {X_s},y \in {Y_s}\}$, where $x$ denotes the token in a sentence ${X_s}$, and $y$ denotes the relation label in ${Y_s}$. The dataset with unseen label is denoted as ${D_u} = \{(x,y)|x \in {X_u},y \in {Y_u}\}$, where ${Y_u}$ denotes the unseen relation set. ${D_s}$ and ${D_u}$ are originally split from the full dataset which defined as $D = \{{S,T,Y}\}$, where *S* denote contained sentences, *T* denote output relation triplets and *Y* denote the contained relation label set. The relation triplet is represented as $t = \{ (y,{e_{head}},{e_{tail}})|y \in Y\} ,t \in T$, where $(y,{e_{head}},{e_{tail}})$ denotes relation label, head entity, and tail entity, respectively. Note that the unseen relation set ${Y_u}$ have no intersection with ${Y_s}$, *i.e*., ${Y_s} \cap {Y_u} = \emptyset$. The seen and unseen relation sets are predefined as $C \in {\mathbb{R}} ^ {n \times (|Y_s| + |Y_u|)}$. We define the zero-shot relation triplet extraction task in a supervised manner by generating synthetic training samples with $\{ {D_s},{Y_s}\}$.

#### Ontological knowledge preliminary

The ontology, denoted as ${\mathscr O} = \{ {\mathscr C},{\mathscr E},{\mathscr D}\}$, is multi-relational ontology graph. where ${\mathscr C}$ represents the concept nodes set, ${\mathscr E}$ is the property edges set that connects the ontology nodes, and ${\mathscr D}$ is the ontology description. In general, the ontology description is a set of RDF triples. The concept nodes in this article take different formats according to the specific domain. For instance, we select the ontology types that are more relevant to entity mention as the logical semantic relation between entity mention and relation labels. As for the connected property edges ${\mathscr E}$, it refers to a link between two concept nodes. We leverage relation property and combine domain-specific properties with RDFS (https://www.w3.org/TR/rdf-schema/) to create edges in the ontology. For example, the knowledge representation {*rte:stateorprovince_of_headquarters*, *rdfs:subClassOf*, *rte: country_of_headquarters*} in Fig. 1 and the relationship {*stateorprovince_of_headquarters*} are the subclasses of relationship {*country_of_headquarters*}, which are denoted as the headquarters of a country and the headquarters of a state or province, respectively. In addition to the structural RDF triplets with structural relationships between concepts, each concept node also contains a textual description in the ontological schema. In this article, we utilize the textual description of ontology knowledge as an auxiliary prompt (details are described in “Knowledge-based prompt temple construction”).

### Knowledge-based prompt temple construction

The process of prompt template construction involves ontology transformation, and the converted text format is more conducive to knowledge injection. The overall generation and extraction process can refer to Algorithm 1.

**Algorithm 1:** Knowledge-based prompt tuning for relation triplet extraction.

**Input:** Training samples *D*_*s*_, Test samples *D*_*u*_, Ontology knowledge *K*_*o*_, $D = {D_s} \cup {D_u}$
**Output:** Multiple relation Triplets *T*_*r*_
**1** Training(*D*_*s*_,*PLM*_*g*_) $\to PL{M_{g,finetune}}$
**2** Training(*D*_*s*_,*PLM*_*e*_) $\to PL{M_{e,finetune}}$
**3** Generating( $PL{M_{g,finetune}} + {\mathrm{Syntax - AwareAttentionNetwork}}({\mathrm{SAAN}}),{K_t},{Y_u}$) $\to {D_{sy}}$
**4** Training( $PL{M_{e,finetune}} + {\mathrm{SAAN}},{D_{sy}}$) $\to PL{M_{e,finished}}$
**5** /*For a detailed description of the SAAN refer to Algorithm 2*/
**6** Predict( $PL{M_{e,finished}},{D_u}$) *∪* Multiple Triplets Decoding (MTD) $\to {T_r}$
**7** /*For a detailed description of MTD refer to Algorithm 3*/
**8** **Return** Multiple relation triplets *T*_*r*_

**Algorithm 2:** Syntax-aware attention network (SAAN).

**Input:** Ontology representation ${\tilde H_o} \in {\mathbb{R}}^{N \times d}$, Relation embedding ${\tilde H_r} \in {\mathbb{R}}^{m \times d}$
**Output:** Integrated embedding $\tilde H_o^{(l)\prime } \in {\mathbb{R}}^{N \times d}$, $\tilde V \in {\mathbb{R}}^{m \times d}$
**1** **Initialize:** Key $\bar K = \{ {\tilde H_r};{\tilde H_o}\}$, $\bar K \in {\mathbb{R}}^{(N + m) \times d}$, Values matrix $\bar V \in {\mathbb{R}}^{m \times d}$;
**2** **for** *hidden embedding* ${\tilde H^{(l)}}$ from *L* layer PLM **do**
**3** Calculate the similarity matrix $S_i^o$
**4** **for** each $W_i^o$ in linear transformation matrices $\{ W_1^o,W_2^o,...,W_N^o\} \in {\mathbb{R}}^{N \times d}$ **do**
**5** $\tilde H_o^{(l)}W_i^o{\bar K^T} \to S_i^o$, $(S_i^o[j,k]\;|\;\forall j,k\;\exists {h_j} \in {\tilde H_o},{h_k} \in \bar K\;and\;{\tilde H_o},\bar K \ne \emptyset )$
**6** $\triangleright\; \bf{Case1}$(update ontology embedding $\bar V$):
**7** **for** each $S_i^o$ in $S^o$, ${S^o} \ne \emptyset$ **do**
**8** Softmax $(S_i^o) \to {\alpha _i}$, $\tilde H_o^{(l)} + [{\alpha _i}\bar V]{W^{o\prime }} \to \tilde H_o^{(l)\prime }$; Saving vector
$\{ \tilde h_{o1}^{(l)\prime },\tilde h_{o2}^{(l)\prime },...,\tilde h_{oN}^{(l)\prime }\} \to \tilde H_o^{(l)\prime },\tilde H_o^{(l)\prime }{\in {\mathbb{R}}^{N \times d}}$
**9** $\triangleright\; \bf{Case2}$ (update relation embedding ${\tilde H_r}$):
**10** **for** $S_i^r\;in\;{S^r},{S^r} \ne \emptyset$ **do**
**11** Calculating attention weight ${\beta _i}$, Softmax $(S_i^r) \to {\beta _i},{\beta _i} \in [0,1]$
**12** Aggregated operation $\bar V^\prime ,[{\beta _1}{\tilde H_r};{\beta _2}{\tilde H_r},...,{\beta _m}{\tilde H_r}] \cdot {W^r} \to \bar V^\prime$
**13** $\triangleright\;{\bf{Initialize}}{W^{v1}},{W^{v2}} \subset {W^o}$, update relation embedding: $\sigma (\bar V^\prime ,{W^{v1}},{W^{v2}}) \to v,v \cdot \bar V^\prime + (1 - v) \cdot \bar V \to \tilde V$
**14** **return** $\tilde H_o^{(l)\prime },\tilde V$
**15** **return** $\tilde H_o^{(l)\prime },\tilde V$
**16** Update hidden vectors in the L-1 layer of PLM

**Algorithm 3:** Multiple triplet decoding (MTD).

**Input:** The synthetic sample sequence *D*_*sy*_.
**Output:** Multiple relation triplets *T*_*z*_.
**1 Initialize:** Initialize special subsequence ${v_{th}} = 0$, $HeadEntity:\emptyset$;
**2** **for** each token in *D*_*sy*_ **do**
**3** Calculating $p({e_{hi}}) = softmax(toke{n_i})$; ${D_{sy}} \ne \emptyset$
**4** **for** $l = 0;l < k;l + +$ **do**
**5** Calculating ${e_{hl}} = {\mathrm{max}}(p({e_{hi}}))$; ${e_{hl}} \to {e_h}$ Obtain the top-k head entities: ${e_h} = \{ {e_{h1}},{e_{h2}},...,{e_{hk}}\}$
**6** **while** $toke{n_i} = = Tail\;Entity:$ **do**
**7** the *j* tail entity: ${e_{tj}} \to p({e_{tj}}|{e_{hi}}) = softmax(toke{n_j})$
**8** **for** $l = 0;l < k;l + +$ **do**
**9** Calculating embedding and saving ${e_{tl}} = {\mathrm{max}}(p({e_{tj}}));{e_{tl}} \to {e_t}$
**10** Obtaining the top-k tail entities ${e_t} = \{ {e_{t1}},{e_{t2}},...,{e_{tk}}\}$
**11** **while** $toke{n_i} = = Relation:$ **do**
**12** the *z-th* relation ${y_z} \to p({y_z}|{e_{tj}},{e_{hi}}) = softmax(toke{n_z})$
**13** **for** $l = 0;l < k;l + +$ **do**
**14** Calculating and Saving ${y_r} = {\mathrm{max}}(p({y_{rz}}));{y_{rz}} \to {y_r}$
**15** Obtaining the top-k relations ${y_r} = \{ {e_{r1}},{e_{r2}},...,{e_{rk}}\}$
**16** Saving Triplets $p({e_{hi}},{e_{tj}},{y_{rz}}) \to p(Triple{t_{i,j,z}})$;
${D_{sy}} = = \emptyset$; $atten({e_{hi}},{e_{tj}},{y_{rz}}) \to {v_{th}},i,j,z \in [0,k];$ $top\;{v_{th}}(Triplets) \to {T_z}$
**17** **return** *T*_*z*_

#### General formulation of prompt template

Let ${X_{in}} = \{ {x_1},{x_2},...,{x_L}\}$ be a input a sentence, where *L* denotes the sequence length and ${x_i}$ denotes the $i\_th$ token in the sentence. Firstly, ${X_{in}}$ is converted into a fixed format sequence ${\tilde X_{in}} = [{\mathrm{CLS}}]{X_{in}}[{\mathrm{SEP}}]$, then we put ${\tilde X_{in}}$ into a PLMs and encode a corresponding hidden vector ${h_k} = \{ {h_{{\mathrm{CLS}}}},{h_1},{h_2},...,{h_k},...,{h_{{\mathrm{SEP}}}}\}$, and ${h_k} \in {\mathbb{R}}^ {k \times d}$. The vanilla fine-tuning approach in prompt learning (Zhang et al., 2021b) utilizes the MLP layer to predict the relation class with an activation function. The parameters in PLM are constantly updated by optimizing the value of the loss function over $P(y|x)$ on the training set. The general practice is to manually design a prompt template ${T_m}( \cdot )$ and label set *V*, and then predict the [MASK] *via* PLMs where the predicted class is contained in the label space. Specifically, the template involves the [MASK] position and the number of added prompt words. For each input sequence ${X_{{\mathrm{in}}}}$, the prompt template aims to map the input sample ${X_{{\mathrm{prompt}}}} = {T_m}({X_{{\mathrm{in}}}})$. Defining the label words by injective mapping $M:M(Y) \to V$ to the specific label class in the vocabulary space, and *V* denotes the label words set. Besides, one or more [MASK] is inserted into the prompt template to predict the label words. The specific prediction form is denoted as $P(y|x) = P([{\mathrm{MASK}}] = M(Y)|{X_{{\mathrm{prompt}}}})$.

In this article, we follow previous studies (Schick & Schütze, 2020) and design a prompt template for zero-shot tasks to generate synthetic training examples that represent the desired relation class. Specifically, to inspire inherent knowledge in the PLM, we formulate ${X_{{\mathrm{prompt}}}}$ containing relation label, ontology knowledge, and the virtual tokens ${w_v}$ with the ${X_{{\mathrm{in}}}}$ is directly tasked with the PLM as follows:

(1) $${X_{{\mathrm{prompt}}}} = [{\mathrm{CLS}}]{X_{{\mathrm{in}}}}[{\mathrm{SEP}}]{T_m}[{\mathrm{SEP}}].$$

**Figure 2:**
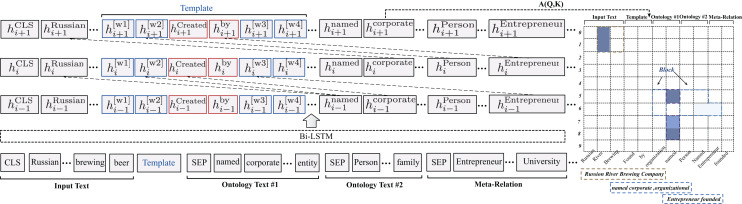
Illustration of span-level knowledge injection. In this instance, $h_i^{text}$ indicates hidden vectors obtained by the BiLSTM. The text in blue and red squares is a virtual token and relation label. The rectangle on the right represents the span-level knowledge matrix, where the dark blue blocks represent the knowledge most relevant to the relation triplets in the input text.

The overall framework of knowledge-based prompt template construction is shown in Fig. 2. Specifically, the external ontology schema as auxiliary knowledge is injected into the prompt template to enhance relation semantics. The primary ontology knowledge includes entity type information and relation scope information. For instance, there are many relation scopes shown in Fig. 1, such as *winery_in_city*, *has_office_in_city*. The ontology schema has rich semantic information, which can assist in knowledge reasoning and decision-making in specific application scenarios. In this article, we mainly utilize it to make up for the missing relation semantics in the prompt template. For the specific format of ontological schema, refer to the Ontology knowledge transformation.

#### Ontology knowledge transformation

The ontology knowledge in the knowledge base has been standardized by RDFS, including five categories of entity class: *rdfs:Class*, relation constraints: *rdfs:domain*, *rdfs:range*, hierarchical structure relationship of parent class and subclass level *rdfs:subClassOf*, *rdfs:subProperty*. To describe the ontology information more clearly, let $h,t,e \in E$ denote the entity variable, ${r_d},{r_p},r \in R$ denote relation variable, and ${c^o},c_{he}^o,c_{te}^o,c_d^o,c_p^o \in {C^o}$ denote meta-type variable in external knowledge base. To take full advantage of the completeness of knowledge representation, we further normalize the entity-relation information by logical rules. Specifically, the standard format is as follows:

– The input sequence’s head entity class and tail entity class have been restrained by the relation knowledge.

$(r,h,t) \wedge (r,{\mathrm{domain}},c_{he}^o) \to (h,{\mathrm{class}},c_{he}^o)$

$(r,h,t) \wedge (r,{\mathrm{range}},c_{te}^o) \to (t,{\mathrm{class}},c_{te}^o)$

– Structured knowledge is valuable for uncovering the implicit relation semantics in the external knowledge base.

$({c^o},{\mathrm{subClassOf}},c_s^o) \wedge (c_s^o,{\mathrm{subClassOf}},c_p^o) \to ({c^o},{\mathrm{subClassOf}},c_p^o)$

$(r,{\mathrm{subPropertyOf}},{r_d}) \wedge ({r_d},{\mathrm{subPropertyOf}},{r_p}) \to (r,{\mathrm{subPropertyOf}},{r_p})$

The additional capabilities of knowledge reasoning in ontological knowledge can uncover the deeper implicit semantics, which assists in generating desire relation instances in a zero-shot setting application scenario.

The ontology representation is denoted as ${\mathscr O} = \{ {\mathscr C},{\mathscr E},{\mathscr D}\}$. As the above mentioned, ontology knowledge contains detailed description information, which are lexically meaningful information of concepts, and it also can be denoted as a triplet with properties, *e.g*., *rte:domain* as shown in Fig. 1. We utilize the entity type as an external knowledge injection. Moreover, to intuitively utilize the lexically semantic in the ontology schema, we first conduct ontology transformation before injecting the prompt template. Specifically, we extract all concept nodes with the assistance of an external ontology schema and then convert this ontology knowledge into the original text descriptions.

For real-world relation triplet, entity class *rdf:Class* semantic information is generally constrained by the various relations in ontology schema. In this article, the MUC (Vilain et al., 1995) is used to define entity concept. The final prompt template is defined as [CLS] ¡ InputText¿ [SEP] ¡ Template¿ [SEP] ¡ OntologyText¿ [SEP] is shown in Fig. 1. Note that the relation label as the internal prompt in ¡ Template¿, we also define several special tokens^1^ 1The number of tokens is a hyper-parameter. to build in the ontological edges (*e.g*., *rdfs:instance_of*), where [w1]–[w4] as the virtual tokens on both sides of the relation label. The virtual tokens are the words with certain relational semantics generated from the relation label, which can assist the model in learning the most relevant entity class for prompt text. Moreover, we construct placeholders in the ¡OntologyText¿ and fill it with text descriptions. Besides, we leverage the path between entity pairs from the ontology as meta-relation text to enhance the ¡OntologyText¿ as shown in Fig. 1.

Previous study (Liu et al., 2020) indicated that superabundant external knowledge injection might introduce extra noise, resulting in poor performance. To alleviate this issue, we propose utilizing the span-level attention matrix to weaken the effects of irrelevant external knowledge. The specific schematic diagram is shown in Fig. 2. Specifically, given the input sequence ${X_{in}} = [{x_1},{x_2},...,{x_L}]$ with *L* tokens, to constrain the impacts of external knowledge injection on the input text, a banded mask matrix $M(R) \in {\mathbb{R}}^{N \times N}$ is defined. *R* denotes the predefined constant radius representation centered at each word of the context sequence, and the total context size of each word can be calculated as $(2R + 1)$. In the specific Transformer network architecture (Vaswani et al., 2017), the attention weight matrix is ahead of the Softmax classifier. Therefore, we redefined the mask matrix as follows:

(2) $$M{(R)_{i,j}} = \left\{ {\matrix{{\!\!0\qquad {x_i},{x_j} \in {X_{in}}\;{\mathrm{and}}\;|i - j| \le R} \cr {\!\!\!\!0\qquad {x_i},{x_j} \in {X_o}\;{\mathrm{and}}\;|i - j| \le R} \cr {0\qquad {x_i} \in {X_{in}},{x_j} \in {X_o}\;{\mathrm{and}}\;i = {p_k}} \cr { - \infty \quad\, {\!\!\mathrm{else}}\,\,\,\quad\quad\quad\quad\quad\quad\quad\quad\quad\quad}\cr } } \right.$$

where ${x_i},{x_j}$ are tokens from input ${X_{in}}$ and ontology description text ${X_o}$, respectively. Note that $- \infty$ denotes obstructing token $i$ from approaching token $j$, and 0 does the exact opposite, encouraging them to attend to each other. Moreover, ${x_i}$ can approach ${x_j}$ condition to both of them pertaining to ${X_j}$, or the ontology description ${X_o}$. Another situation is that token ${x_i}$ is an entity mention, and ${x_j}$ comes from the ontology description ${X_o}$. The ${p_k}$ denotes the position of the entity span in the input text.

Given the input text and the ontology description text, we first convert the tokens into embedding representations and then feed them into a deep neural network bidirectional LSTM (Bi-LSTM) (Melamud, Goldberger & Dagan, 2016) to obtain the hidden vector $H = \{ {H_{in}};{H_o}\}$, where ${H_{in}}$, ${H_o}$ indicate the input hidden vector and ontology hidden vector, respectively, and; denotes the connect operation. Besides, ${H_{in}} \in {\mathbb{R}}^{L \times d}$ and ${H_o} \in {\mathbb{R}}^{N \times d}$, *N* is the length of tokens in ontology text, and $d$ is the dimension of hidden state in bidirectional LSTM. To leverage the self-supervised attention in Transformer (Vaswani et al., 2017), the vector *H* is projected into different subspaces to obtain the queries vector $Q \in {\mathbb{R}}^{(L + N) \times d}$, keys vector $K \in {\mathbb{R}}^{(L + N) \times d}$, and the value vector $V \in {\mathbb{R}}^{(L + N) \times d}$ with linear transformation. Then we calculate the attention weight matrix *A* by using the mask matrix $M{(R)_{i,j}}$ as follows:

(3) $$A = {\mathrm{MASK}}\_{\mathrm{ATT}}(Q,K) = {\mathrm{SoftMax}}(Q{K^T} + M(R)),$$

where $i,j \in \{ 1,2,...,(L + N)\}$, and $A \in {\mathbb{R}}^{(L + N) \times (L + N)}$. Therefore, when the value of the mask matrix is negative infinity, the attention weight ${A_{i,j}}$ tends to zero, for ${x_i}$, the information of ${x_j}$ is ignored. Moreover, it assists in filtering out irrelevant noise knowledge with a static working scope for all input sequences, and the injection process of the attention mask matrix is shown in Fig. 2.

The overall framework of the span-level matrix knowledge injection process is shown in Fig. 2. First, the input text and ontology knowledge are fed into a BiLSTM network and converted the text sequence into a hidden vector $h_i^{text}$, then filtering on irrelevant ontology knowledge with the mask matrix $A(Q,K)$. Specifically, when the value of the mask matrix is negative infinity, ${A_{i,j}}$ becomes zero and is calculated by Eq. (3). Therefore, for token ${x_i}$, the token ${x_j}$ from ontology text is ignored. For each token in the input text, only the previous *R* tokens and the following *R* tokens are attended to. In this way, the ontology knowledge related to entity ${e_i}$ and within the range of $(2R + 1)$ is reserved, and knowledge beyond the range is filtered. The entities mentioned in Fig. 2 are depicted in dark color, and the ontology knowledge retained is shown in the blue and grey rectangle below.

To filter out noise effectively and learn token-specific knowledge adaptively, we utilize a feed-forward neural network (FFN) (Schmidhuber, 2015) to determine the different context ranges of each token. Therefore, each token can obtain an apposite context size in this way. We further define a learnable token radius vector $\tilde r \in {\mathbb{R}}^{L + N}$ to denote the represented context radiuses. For the $i - th$ token, each value ${\tilde r_i} \in \mathbb{R}$ represents the learned textual radius and its value between 0 and *R*. The radius vector $\tilde r$ is defined as follows:

(4) $$\tilde r = \sigma ((H{W_r} + {b_r}){W_{r^\prime }} + {b_{r^\prime }}) \cdot R$$

(5) $$\sigma (x) = {1 \over {1 + {\mathrm{exp}}( - x)}}$$

where ${W_r} \in {\mathbb{R}}^{2d \times d}$, ${W_{r^\prime }} \in {\mathbb{R}}^d$, ${b_r},{b_{r^\prime }} \in {\mathbb{R}}^{L + N}$ are learnable parameters. $\cdot$ denotes dot product operation, and $\sigma ( \cdot )$ is the sigmoid activate function ranging from zero to one. Note that there is no ontology information when $\tilde r$ is zero. Therefore, to ensure at least one token is utilized to represent the ontology knowledge, the radius vector $\tilde r$ is further revised as below:

(6) $$\tilde r = {\bf{1}} + \sigma ((H{W_r} + {b_r}){W_{r^\prime }} + {b_{r^\prime }}) \cdot (R - 0.5)$$

where ${\bf{1}} \in {\mathbb{R}}^{L + N}$ is a tensor with each value equal to one. Thus, we can obtain the mask matrix with each row by utilizing the learned textual radius. Then, each token is constrained into different span sizes according to the attention weights (shown in the dark-colored blocks in Fig. 2). To obtain the integer attention weight matrix $\tilde A$, we leverage the end-to-end manner to train the model and do not lose the differentiability. Specifically, for the $i - th$ token, the attention weight matrix $\tilde A$ is defined as follows:

(7) $${\tilde A_{(i,)}} = \sum\nolimits_p {{\mathrm{softmax}}} ({S_i} + {\mathrm{M}}{(p)_i}) \cdot \tilde K({\tilde r_i},p)$$

(8) $$\tilde K(a,b) = {\mathrm{max}}(0,1 - |a - b|)$$

where ${\tilde A_{(i,)}}$ denotes the $i - th$ row in matrix $\tilde A$. The learnable attention weight matrix has a different token-level context radius for all tokens in an input sequence. ${S_i} = Q{K^T}$ denotes the attention score matrix of the $i - th$ row, and $p$ counts the number of integral textual radius in the input sequence. ${\mathrm{M}}{(p)_i}$ denotes the mask matrix with textual radius value is $p$. $\tilde K( \cdot )$ is the Relu activation function with a faster convergence speed and can effectively relieve the vanishing gradient problem.

### Relation data generation

PLMs are implicitly capable of zero-shot generalization based on their general and large-scale pre-training. Therefore, we utilize the external ontology knowledge and the relation label as prompts to generate structural training data by conditioning on the target unseen relation labels. Specifically, the PLM BERT (Devlin et al., 2018) combined with the syntax-aware attention network as the language model. The output prompt is denoted as $\tilde H = \tilde AH$, where $\tilde H \in {\mathbb{R}}^{(L + N) \times d}$ as the input of the language model.

#### Syntax-aware attention network

To enhance the fusion of relation semantic and external ontology knowledge, we add a syntax-aware attention network (SAAN) in the last $k$ layer of PLM. Specifically, the external knowledge representation and relation label embedding are joint as slot entries in the syntax-aware network. In addition, we also designed the read and write operators well, allowing relation labels to attend to the highly correlated external ontology knowledge representation. Besides, we initialize the input of the attention network by ontology description and meta-relation embedding representation. Concretely, the length of ontology knowledge tokens is fixed as *N*, and the embedding representation is denoted as ${\tilde H_o} = \{ {\tilde h_{o1}},{\tilde h_{o2}}...,{\tilde h_{oN}}\}$, and the relation label embedding is denoted as ${\tilde H_r} = \{ {\tilde h_{r1}},{\tilde h_{r2}}...,{\tilde h_{rm}}\}$, $m$ is the length of relation token. Finally, composing the representation matrix of keys by connecting operations $\bar K = \{ {\tilde H_r};{\tilde H_o}\}$, and further gain the representation matrix of values that denoted as $\bar V = \{ {\bar h_{o1}},{\bar h_{o2}},...,{\bar h_{om}}\}$ by the linear operation.

The prompt vector $\tilde H$ is converted into token representation embedding ${\tilde H^{(l)}}$ after the $l - th$ layer of PLM. Furthermore, we carefully design a multi-dimensional read operation to capture this associated semantic knowledge in the SAAN. Specifically, given a linear transformation matrices $\{ W_1^o,W_2^o,...,W_N^o\} \in {\mathbb{R}}^{N \times d}$, where *N* denotes the sequence length in ontology text, and generating the multiple semantic similarity matrices $\{ S_1^o,S_2^o,...,S_N^o\} \in {\mathbb{R}}^{N \times d}$ between ontology tokens and relation representation within the attention network, then output the vector representation by an aggregate function. The specific semantic vector $S_i^o$ is defined as follows:

(9) $$S_i^o = \tilde H_o^{(l)}W_i^o{\bar K^T}$$

where $W_i^o$ is the learnable parameter matrix, and $S_i^o(j,k)$ denotes semantic similarity between $j - th$ token and $k - th$ token in the visualization matrix. We further update the token representation by utilizing the semantic similarity matrix with an aggregate operator, and the calculation formula is as follows:

(10) $$\tilde H_o^{(l)\prime } = \tilde H_o^{(l)} + [{\alpha _1}\bar V;{\alpha _2}\bar V,...,{\alpha _L}\bar V]{W^{o\prime }}$$

(11) $${\alpha _i} = {\mathrm{softmax}}(S_i^o)$$

where ${W^{o\prime }} \in {\mathbb{R}}^{N \times d}$ denotes the learnable parameter matrix and ${\alpha _i}$ denotes attention weight that following the values matrix $\bar V$. In this way, the relation label can be complementary with external ontology knowledge to generate more conducive synthetic samples for the unseen relation. After that calculation, the obtained vector representations $\tilde H_o^{(l)\prime }$ are fed into the next layer of PLM to assist in generating synthetic training data, where the integrated knowledge from syntax attention network is beneficial to capture the essential information in the Transformer.

After updating the text representation, we use the attention network to implement the relation label embedding. Specifically, we leverage multi-dimension similarity matrices ${S^r} \in {\mathbb{R}}^{m \times d}$ calculating attention weight distribution $\beta$ with input token representation, and then obtain the new knowledge representation by an aggregated function. The calculation formula is as follows:

(12) $$\bar V^\prime = [{\beta _1}{\tilde H_r};{\beta _2}{\tilde H_r},...,{\beta _m}{\tilde H_r}]{W^r}$$

(13) $${\beta _i} = {\mathrm{softmax}}{(S_i^r)^T}$$

where $\bar V^\prime \in {\mathbb{R}}^{m \times d}$. ${W^r} \in {\mathbb{R}}^{N \times d}$ is learnable parameter matrix. To further fuse relation representation and the ontology embedding, a gated recurrent unit is integrated with the aggregated relation representation, and the updated relation representation calculation formula is as follows:

(14) $$v = \sigma (\bar V^\prime {W^{v1}} + \bar V{W^{v2}})$$

(15) $$\tilde V = v \cdot \bar {V}^\prime + (1 - v) \cdot \bar V$$

where ${W^{v1}}$, ${W^{v2}}$ are learnable parameter matrices with the dimension $d$. The updated relation representation is also fed into the PLM. Thus, relation representation and ontology embedding are fused to aggregate more effective knowledge to assist in the generation of synthetic training samples.

#### Decoding relation triplets

We further propose a generation decoding method in order to improve the zero-shot extraction performance on sentences that contain multiple triplets. The constructed ontology knowledge prompt is denoted as { ${\mathrm{Relation}}\!\!: \!{{\mathrm{y}}_{\mathrm{s}}};\!{\mathrm{Ontology}}\!\!:\!{{\mathrm{X}}_{\mathrm{o}}}$}, and the output text in the form of {Text:T; Relation: ${{\mathrm{y}}_{\mathrm{u}}}$; Head entity: ${{\mathrm{e}}_{{\mathrm{head}}}}$, Tail entity: ${{\mathrm{e}}_{{\mathrm{tail}}}}$}. The hidden feature embedding is denoted as ${\tilde H^{(l)}} = \{ h_1^{(l)},h_2^{(l)},...,h_{L + N}^{(l)}\}$. To further enhance the fusion of relation label and ontology knowledge, we utilize the continual pre-training in the seen dataset. Finally, the conditional probability is represented to predict each generated token as follows:

(16) $$L(x) = \prod\limits_{i,j = 1}^n P ({x_i}|{x_{j < i}})$$

(17) $$P({x_i}|{x_{j < i}}) = {{{\mathrm{exp}}(h_i^T/tm)} \over {\sum\nolimits_{j = 1}^{|V|} {{\mathrm{exp}}} (h_j^T/tm)}}$$

where $n$ denotes the length of the sequence, and $P({x_i}|{x_{j < i}})$ represents the conditional probability that predicts tokens of the synthetic training samples in the position $i$, ${h_i}$ is the hidden vector. To keep the generated synthetic data as diverse as possible, we utilize a prediction model with temperature $tm$ (Hinton, Vinyals & Dean, 2015) on the vocabulary size *V*, and the loss function also changes accordingly. During training, the decoding layer predicts target token ${x_i}$, which encourages the decoder to generate target tokens that are close to the seen relation tokens ${y_i}$ by the probability distribution $P({x_i}|{x_{j < i}})$. A squared error loss function is utilized as follows to update the weight parameter:

(18) $${{\cal L}_{loss}} = {1 \over {|V|}}\sum_{j = 1}^{|V|} {\sum_{i = 1}^{|V|} {(P(} } {x_i}|{x_{j < i}}) - {y_i}{)^2}.$$

To alleviate error decoding, we utilize the squared error loss function rather than cross-entropy loss. The format of the generated structured synthetic instances are represented as {Context:, Head entity:, Tail entity:}, where the unseen relation is contained in the *Context*. We discard the error sample if an entity is not found in the generated sequence and continue to search until reach a fixed number of synthetic samples.

### Multiple relation triplets extraction with multiple triplets decoding

**Figure 3:**
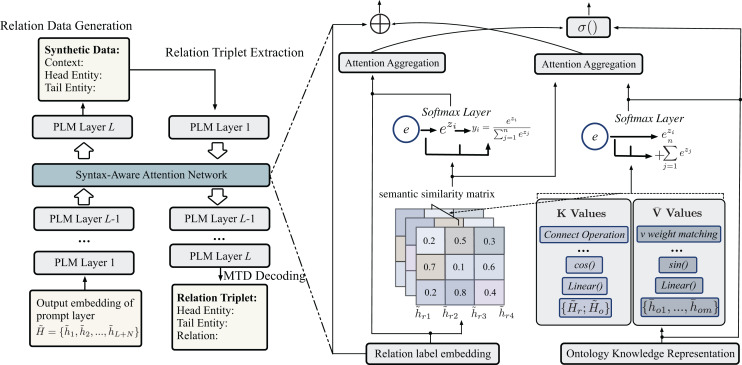
The illustration of relation data generation and relation triplet extraction. The model encodes the input text and prompt template using PLM, and the SAAN is added in the last L layers to fuse embedding representations. The hidden embedding from the prompt layer generates synthetic data during the Data Generation phase, and then the model with generated synthetic data to output the relation triplets during the relation triplet extraction phase. The SAAN (dark green on the left of the figure) is utilized in both stages, and the detailed calculation process of the SAAN is described on the right of this figure.

The synthetic training samples generated for unseen relations are employed to train the model during the relation triplet extraction phase. Subsequently, a relation extractor is trained utilizing the pre-trained language model (PLM) BERT to extract multiple relation triplets. An overview of the overall framework is depicted in Fig. 3. Specifically, fine-tuning is first conducted on the seen dataset ${D_s}$ and then further tuning is performed on the model ${M_e}$ on the synthetic training samples. Finally, the trained model is utilized to predict the unseen relation triplets ${T_e}$. In addition, the model also fully utilizes the SAAN mentioned in Syntax-aware attention network(SAAN), enhancing its design sophistication to tackle various tasks effectively. The input text $Context:s$ is fed into the middle layer of the relation extractor. The output sequence format is represented as Relation: ${{\mathrm{y}}_{\mathrm{u}}}$, Head Entity: ${{\mathrm{e}}_{{\mathrm{head}}}}$, Tail Entity: ${{\mathrm{e}}_{{\mathrm{Tail}}}}$. Please refer to the structure diagram depicted in Fig. 3 for visual representation. The standard decoding layer of PLMs excels at performing decoding and greedy generation tasks flawlessly. Additionally, the language model can independently predict multiple relation triplets from an input sentence $s$, and it can decode output sequences without requiring any initialization. In the event of decoding a faulty entity or relationship, it will be discarded and treated as a null prediction.

To improve the zero-shot triplet extraction performance, the multiple triplets decoding algorithm (MTD) was proposed based on the beam search algorithm (Wang et al., 2021). The aim of the algorithm is to search multiple triplets that interest the unseen relation in a sentence *via* an attention score. Because the generated synthetic data only comprises a relation triplet, standard models typically underperform within the current framework. This is because they often assume that a training instance must include multiple triplets when conducting triplet prediction. The conventional approach based on beam search may try to alleviate this issue but cannot migrate to the large-scale relation triplet extraction task, and it is not easy to extract multiple triplets at once in a sentence. Hence, to address this limitation, the Multiple Triplets Decoding algorithm (MTD) was proposed, which enables the extraction of multiple relation triplets simultaneously from a single sentence.

The essential concept of the MTD algorithm is to frame this extracted process as a conditional search task. In general, given an input sentence into the PLM extractor, it can output a sequence through autoregression and greedy decoding. Nevertheless, MTD can deliver multiple sequences at once, and each of them corresponds to various candidate triplet sets. The specific description is shown in Algorithm 3. Subsequently, the attention mechanism is used to calculate a similarity threshold to control the final output number. The main approach involves determining multiple output sequences from candidate head entities, tail entities, and relation labels through a probability distribution model. Specifically, the sentence in Fig. 1 can be taken as an example. The subsequence {Head Entity:} can be denoted as the initiating sequence, and the following token can be denoted as the generated head entity such as *Brewing Company* calculated by the softmax probability model $p({e_{hi}}|{\mathrm{Head}})$, where ${e_{hi}}$ denotes the $i - th$ possible head entity. Moreover, the attention mechanism is utilized to calculate an attention score, which is used to identify the next generated tokens of the entity sequence with the largest attention weight.

The model decodes the top-k sequence based on the highest top-k probability $p({e_{hi}}|{\mathrm{Head}})$ and generates top-k entities rather than greedily decoding the whole sequence. Subsequently, this sequence continues to decode until generating the marked token {Tail Entity:}, and the next predicted token is the first token of the tail entity. The probability model of the first token in $j - th$ tail entity is represented as $p({e_{tj}}|{e_{hi}})$, which is conditioned to the generated head entity. Therefore, for each head entity, there are also top-k tail entity sequences based on the top-k highest conditional probability $p({e_{tj}}|{e_{hi}})$. In this way, the sequence greedily decodes in the same way until it generates the unique token {Relation:}, and then the generated next token becomes the first token of $z - th$ relation by means of the probability prediction model $p$ such as *Created by* in Fig. 1. In turn, the $k$ relation condition is determined based on the output entity pairs with the top-k probability $p({y_z}|{e_{hi}},{e_{tj}})$. For each instance, the decoding process is greedy until the end token is generated. The specific algorithm pseudocode is shown in Algorithm 3, and the algorithmic inference process can be calculated as follows:

(19) $$\eqalign{p({\mathrm{triple}}{{\mathrm{t}}_{i,j,z}})& = p({e_{hi}},{e_{tj}},{y_z}) \\& = p({y_z}|{e_{hi}},{e_{tj}}) \cdot p({e_{tj}}|{e_{hi}}) \cdot p({e_{hi}}).}$$

According to the Formula (19), the conditional probability model does not directly attend to context due to the existence of special tokens in the generated template. Additionally, the formula does not include the input sentence because it is treated equally when outputting multiple triplet sequences, which is only used for one sample. Consequently, there are different triplet sequences corresponding to ${k^3}$ for each instance. Therefore, a threshold over the validation ${{\mathrm{F}}_{\mathrm{1}}}$ metric (Powers, 2020) is calculated to select the suitable output triplets. The specific parameter settings can be found in Hyperparameters study.

The approach of MTD can achieve a directly calculated probability of each output triplet by utilizing $p({\mathrm{triple}}{{\mathrm{t}}_{i,j,z}})$, and can also restrict the number of output triplets with a specific threshold value compared to another extract approach (Wang et al., 2021). Further, it utilizes the structured text format for greedy decoding to achieve a one-to-one mode. Additionally, the zero-shot setting relation triplet extraction allows the relation extraction model to extract multiple triplets simultaneously despite training on synthetic samples with a single triplet each.

## Experiments and results

In this section, we evaluate the KBPT on four public datasets. They are FewRel, Wiki-ZSL, NYT, and TACRED-Revisit, respectively. We conduct exhaustive experiments for the zero-shot setting scenarios to demonstrate the effectiveness of the proposed model. The detailed description and analysis will be divided into chapters below.

### Datasets and evaluation metrics

To evaluate the performance of the KBPT in the zero-shot setting scenario, we re-split these public datasets for training, validation, and testing set according to a specific ratio.

**FewRel** is human-annotated, and each relation corresponds to 700 samples. Please refer to Table 1 for detailed statistics. The initial assessment of this benchmark is for the low-resource relation classification (RC). However, to meet the needs of the zero-shot setting, we reset the ratio of seen and unseen relations to 1:1, *i.e*., 40 seen and 40 unseen relations, ensuring disjoint relation categories in training and test sets. Specifically, To include seen relation instances in the test set, we set each seen relation in the training set to contain 500 cases and the remaining 200 samples in the test set. In the test set, 40 unseen relations, each relation corresponds to 700 instances. Note that we fine-tuned our model on the Wiki-ZLS validation set and transferred the model to the FewRel directly without tuning parameters.

**Table 1:** Datasets statistic. The statistic of “Sentence Length” here means the average number of words in a sentence.

Dataset	Samples	Entities	Relations	Sentence length
FewRel	56,000	72,954	80	24.95
Wiki-ZSL	94,383	77,623	113	24.85
NYT	118,000	170,000	53	25.60
TACRED-Revisit	91,467	–	40	24.01

**Wiki-ZSL** is constructed by distant supervision over Wikipedia articles from the Wikidata knowledge base. To meet the requirements of the zero-shot setting, we follow the same processing procedure of Chen & Li (2021). Specifically, we randomly select a certain amount of relations as the novel relation and the remaining labels as seen relation during the training phase. For better contrast, we set the different sizes of the unseen label as $q$ and want to identify more unseen relation labels. However, different from Chia et al. (2022), we set $q \in \{ 5,10,15\}$ in our experiment. To reduce data noise, we repeat the label selection process six times to divide it into different data blocks with varying random seeds. First, we predefine 10 seen relations in the validation set and the remaining seen relations in the training set. Then, for each data block, the instances belonging to the new relation label in the test set and the seen relations are in the validation and training set. Note that the validation set is used for fine-tuning hyperparameters and early stopping. In this way, our proposed model achieves the zero-shot setting in which the relation labels in training, validation, and test sets belong to disjoint label sets.

**NYT** The NYT dataset is constructed with the relations from aligning Freebase by distant supervision over the New York Times crops (NYT). It is a noisy and uneven distribution dataset with 53 relation labels. We divide 15 seen relation labels as the training set, and each relation label has 7 k sample instances. Besides, we divide 10 seen relation labels as the validation set, and each relation label carries 1 k data samples. The rest of the relation labels and sample instances in the test set constitute the unseen relation samples. Generally, the relation labels are 15, 10, and 28, respectively, and the data samples are 105, 10, and 3 k in training, validation, and test set. Hence, we achieve the zero-shot setting over this dataset.

**TACRED-Revisit** TACRED (Zhang et al., 2017) is a large-scale dataset for relation extraction, consisting of 106 k instances of crowd-sourcing from the TACKBP4 and 42 relation categories (the particular relation label *no_relation* is contained). The original dataset consisted of more than 68 k instances for training, 23 k for validation, and 15 k for testing. The recently released TACRED-Revisit dataset corrects many mislabels in the TACRED dataset. Hence, we adopt the TACRED-Revisit dataset to conduct experiments and re-partition this dataset to meet the requirements of the zero-shot setting. Specifically, we divided 20 seen relation labels in the training set corresponding to 45 k sample instances, 10 seen relation labels in the validation set corresponding to 22 k sample instances, and the rest as unseen relation labels and corresponding sample instances in the test set. In general, it achieves the requirements of the zero-shot setting.

Since our main target is to extract multiple triplets, the evaluation results in this study are for the instances that contain single and multiple triplets separately. To the evaluation metric of multiple triplets extraction, we follow the typical relation extraction metric micro ${{\mathrm{F}}_{\mathrm{1}}}$ score, precision (Prec.), and recall (Rec.). The standard micro accuracy (Acc.) is utilized for single triplet extraction because it only contains one possible triplet in each sentence. These formulas are calculated as follows:

(20) $${\mathrm{Prec}} = {{{\mathrm{TP}}} \over {{\mathrm{TP}} + {\mathrm{FP}}}},{\mathrm{Rec}} = {{{\mathrm{TP}}} \over {{\mathrm{TP}} + {\mathrm{FN}}}}$$

(21) $${\mathrm{Acc}} = {{{\mathrm{TP}} + {\mathrm{TN}}} \over {{\mathrm{TP}} + {\mathrm{TN}} + {\mathrm{FP}} + {\mathrm{FN}}}}$$

(22) $${{\mathrm{F}}_{\mathrm{1}}} = 2 \cdot {{{\mathrm{TP}}} \over {2 \cdot {\mathrm{TP}} + {\mathrm{FP}} + {\mathrm{FN}}}} = 2 \cdot {{{\mathrm{precision}} \cdot {\mathrm{recall}}} \over {{\mathrm{precision}+ {\mathrm{recall}}}}} $$

where ${\mathrm{TP}}$ denotes the actual positives are correctly predicted positives that are called true positives, ${\mathrm{FP}}$ is called false positives, denotes actual negatives that are wrongly predicted positives. ${\mathrm{TN}}$ is called true negative, which denotes the actual negative and is correctly predicted. ${\mathrm{FN}}$ denotes the actual positive that is wrongly predicted negative and is called the false negative.

### Hyperparameters study

For the relation generating stage in Main experimental results, the PLM ${\mathrm{BER}}{{\mathrm{T}}_{{\mathrm{BASE}}}}$ (Devlin et al., 2018) is utilized, which comprises 110 M parameters. To extract multiple triplets more efficiently, the PLM ${\mathrm{BER}}{{\mathrm{T}}_{{\mathrm{LARGE}}}}$ (Devlin et al., 2018) was adopted, which has 340 M parameters in the triplet extraction stage. All the implementations of the PLM were sourced from the Transformers package (wolf2020transformers). In both of these two phases, all fine-tuning operators are conducted on the complete training datasets with five epochs, and early stopping refers to the loss on the validation sets. The learning rate is a crucial parameter, and experimental results suggest that models of different sizes perform optimally with varying learning rates. Therefore, different intervals were employed corresponding to the various model sizes. Specifically, the stochastic gradient descent (SGD) (Ketkar, 2017) algorithm is utilized to select optimal learning rate $\alpha$ among $\{ 2e - 4,5e - 4,3e - 2\}$ for ${\mathrm{BER}}{{\mathrm{T}}_{{\mathrm{BASE}}}}$, and $\{ 5e - 5,2e - 4,3e - 4\}$ for ${\mathrm{BER}}{{\mathrm{T}}_{{\mathrm{LARGE}}}}$ with a linear warmup for the first 10% steps. In general, small models prefer a larger learning rate. Further, to avoid model overfitting, the weight decay was set as $1e - 2$. The model sourced from the best efficiency over the validation set was saved for the test. For the detailed hyper-parameter settings, refer to Table 2.

**Table 2:** Details of hyper-parameters setting.

Parameter	Alternative values	Final value
Manually tuned		
Dataset split (train-test)	{70–20%}{60–20%}	{70–20%}
Hidden layer dimension $d$	{100, 200, 300}	200
Hidden embedding dimension	Up to 768	768
Maximum sequence length	Up to 512	512
Pre-trained language model	{none, GPT-2, RoBERTa,BERT}	BERT
Tuned with KBPT		
Epochs	{5, 10, 15, 20, 25}	10
Batch size	{4, 16, 32, 64, 128, 256, 512}	64
Warmup ratio	{0.1, 0.15, 0.2, 0.3}	0.1
Initial learning rate $\alpha$	{ ${\mathrm{5e - 5}}$, ${\mathrm{5e - 4}}$, ${\mathrm{3e - 4}}$, ${\mathrm{3e - 3}}$, ${\mathrm{3e - 2}}$, ${\mathrm{2e - 4}}$ }	${\mathrm{3e - 3}}$
Weight decay	{ ${\mathrm{1e - 2}}$, ${\mathrm{1e - 3}}$, ${\mathrm{2e - 2}}$, ${\mathrm{2e - 3}}$, ${\mathrm{2e - 4}}$}	${\mathrm{1e - 2}}$
Training dropout probability	{0.1, 0.2, 0.3, 0.4, 0.5, 0.6, 0.7}	0.2
Network optimizer	{SGD, Adagrad, Adadelta, Adam, Nadam, Adamax}	SGD
Activation function	{Linear, Sigmoid, TanH, SoftMax, ReLU, ELU, SeLU}	{Sigmoid, SoftMax, ReLU}
Loss function	{Mean squared error, Mean absolute error, Categorical crossentropy, Sparse categorical crossentropy, Squared hinge}	Mean squared error
Generating sampling top-k	{20, 30, 40, 50, 60}	50
Generating samples each relation label	{200, 300, 400, 500, 1,000}	300
Multiple triplet decoding Top-n branches $p$	{2, 4, 6, 8}	4
Multiple triplet decoding threshold $t$	{−0.912, −2.912, −3.912, −4.912, −5.912}	−0.912

All experiments were conducted on the hardware infrastructure of 2 NVIDIA GeForce RTX 3090 GPUs with a batch size of 64. A certain number of synthetic samples were produced for each unseen relation label at the stage of generating, and the synthetic relation samples were used to supervise the model to extract multiple relation triplets further. Moreover, the hyper-parameters were tuned on the validation set of Wiki-ZSL with 10 seen relation labels. Specifically, the different values of $\{ \hbox{250, 300, 500, 1,000, 2,000\}}$ were considered for the number of generated synthetic samples. Additionally, the value of the threshold of MTD was also tuned. Specifically, sixty equally spaced values were set between the output scores of multiple candidate triplets over the validation set. To conserve computing resources, a fixed value for the number of branches in each stage was established, and it was not adjusted as a hyperparameter. For specific evolvable hyper-parameter settings, refer to Table 2. The remaining experiment section mainly describes the experimental results, comparison methods, specific implementation process for multiple triplet extraction, and further discussion and analysis of experimental results. Experimental insights and results are primarily present in the form of graphs.

### Main experimental results

In this subsection, we report specific experimental results and provide possible insights for our proposed model. The best result is highlighted in boldface in the corresponding tables.

#### Baseline methods

Based on the particularity of the zero-shot setting, two baseline models were designed for comparison with the proposed approach. First, for Wiki-ZSL and FewRel, a strategy similar to RelationPrompt was adopted, denoted as “NoSyn,” which does not utilize generated synthetic samples for training. The reason is that the model did not fine-tune the synthetic training data during the extraction stage in all experiments. During the test phase, the model was constrained to generate the unseen relation label by fine-tuning. Second, the existing triplet extraction model TableSequence (Wang & Lu, 2020) was compared with the proposed method. Experiments were conducted on synthetic training data using supervised learning, as TableSequence cannot perform in the zero-shot setting.

The proposed model was compared with DEEPEX (Wang et al., 2021), REDN (Li & Tian, 2020), and ZSLRE (Gong & Eldardiry, 2020) on the NYT dataset. The choice of DEEPEX for comparison is motivated by its alignment with the present research objectives. DEEPEX’s broader research scope and the design of a unified framework for zero-shot information extraction make it a relevant point of comparison. The reported results for DEEPEX on NYT in Table 3 were re-implemented, as their original evaluation pertains to open information extraction. Additionally, the reported results for REDN and ZSLRE were directly copied from the initial references. Notably, REDN was designed for relation classification, hence only the pre-existing results were included in the experiment for a fair comparison. For the TACRED-Revisit dataset, SOTA models were adopted for comparison in a low-resource setting. Specifically, AdaPrompt-tuning (Chen et al., 2022b) served as the reference standard, and $K = \{ 8,16,32\}$ instances were set for training in few-shot scenarios.

**Table 3:** Results of different models with the zero-shot setting on NYT (%). Our reimplementation is marked with an asterisk (*). The best result is highlighted in boldface.

Model	Pre	Rec	${{\mathrm{F}}_{\mathrm{1}}}$
CDNN	46.40	52.70	45.80
REDN	95.10	94.00	94.60
ZSLRE	**98.10**	97.90	97.60
DEEPEX*	90.34	81.15	85.50
KBPT (our model)	97.34	**98.67**	**98.00**

#### Results on Wiki-ZSL and FewRel

From the experimental data in Table 4, four conclusions could be drawn. Firstly, the unstable precision-recall ratio of these baselines resulted in poor ${{\mathrm{F}}_{\mathrm{1}}}$ score performance for multiple triplet extraction. Secondly, the substantial gap in ${{\mathrm{F}}_{\mathrm{1}}}$ score between NoSyn and KBPT indicates the critical importance of utilizing synthetic training samples during the generating phase for zero-shot settings, as the ${{\mathrm{F}}_{\mathrm{1}}}$ score improved by up to 14.65% (from 7.54% to 22.19%). This also highlights the effectiveness and stability of synthetic data. Thirdly, the generation of multiple triplets was non-trivial due to the presence of only a single triplet in the synthetic training data during the extracting phase. Thus, TableSequence performed considerably worse in ${{\mathrm{F}}_{\mathrm{1}}}$ score compared to RelationPrompt and the proposed method. Although TableSequence can also extract multiple relation triplets through special design, its premise is that the training data may contain multiple triplets in an instance. Fourthly, the experimental results in ${{\mathrm{F}}_{\mathrm{1}}}$ score and the positive differences between RelationPrompt and KBPT for multiple triplet extraction confirm the superiority of KBPT. Further, all ${{\mathrm{F}}_{\mathrm{1}}}$ scores for multiple triplet extraction in KBPT outperformed RelationPrompt, indicating the robust ability of the proposed model to leverage implicit knowledge obtained from external ontology knowledge.

**Table 4:** Results(%) for zero-shot setting relation triplet extraction on Wiki-ZSL and FewRel datasets. The numbers in parentheses are the main differences between RelationPrompt and KBPT (our model) on different metrics. The best result is highlighted in boldface.

Unseen labels (m)	Model	Single triplet		Multiple triplets					
		Wiki-ZSL	FewRel	Wiki-ZSL			FewRel		
		Acc	Acc	Pre	Rec	${{\mathrm{F}}_{\mathrm{1}}}$	Pre	Rec	${{\mathrm{F}}_{\mathrm{1}}}$
5	TableSequence	14.47	11.82	**43.68**	3.51	6.29	15.23	1.91	3.40
	RelationPrompt	16.64	22.27	29.11	31.00	30.01	20.80	**24.32**	22.34
	NoSyn	11.23	20.32	19.61	**48.57**	27.94	14.29	16.46	15.30
	KBPT (our model)	**17.85 (+1.21)**	**24.19 (+1.92)**	32.45 (+3.34)	31.64 (+0.64)	**32.04 (+2.03)**	**23.15 (+3.07)**	23.13 (−1.09)	**24.28 (+1.94)**
10	TableSequence	9.61	12.54	**45.31**	3.57	6.4	**28.93**	3.60	6.37
	RelationPrompt	16.48	23.18	30.20	32.31	31.19	21.59	**28.68**	24.61
	NoSyn	17.85	20.13	20.12	21.59	20.83	15.72	24.14	19.04
	KBPT (our model)	**20.45 (+3.97)**	**26.58 (+3.40)**	32.47 (+2.27)	**33.69 (+1.38)**	**33.17 (+1.98)**	24.35 (+2.76)	27.28 (−1.40)	**26.46 (+1.85)**
15	TableSequence	9.20	11.65	**44.43**	3.53	6.39	19.03	1.99	3.48
	RelationPrompt	16.16	18.97	26.19	**32.12**	28.85	17.73	23.20	20.08
	NoSyn	19.03	20.97	15.78	28.81	20.39	10.83	5.78	7.54
	KBPT (our model)	**20.31 (+4.15)**	**22.46 (+3.49)**	32.15 (+5.96)	29.39 (−2.73)	**30.74 (+1.89)**	**19.61 (+1.88)**	**25.55 (+2.35)**	**22.19 (+2.11)**

**Figure 4:**
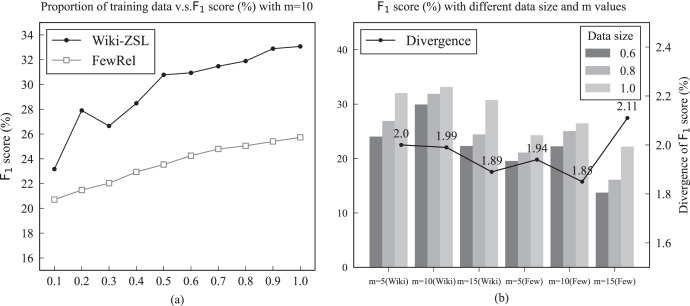
Left (A) results of KBPT with the number of unseen labels ${\mathrm{m = 10}}$ and abscissa scales represent different training data ratios on the Wiki-ZSL and FewRel datasets. Right (B) results of different data sizes and varied m values on the Wiki-ZSL and FewRel datasets. Ordinate scales represent the ${{\mathrm{F}}_{\mathrm{1}}}$ score and the divergence of ${{\mathrm{F}}_{\mathrm{1}}}$ score between KBPT and RelationPrompt, respectively. The three columns represent the corresponding ${{\mathrm{F}}_{\mathrm{1}}}$ score with different m values and data sizes over the two datasets. The broken line represents the divergence of ${{\mathrm{F}}_{\mathrm{1}}}$ score between KBPT and RelationPrompt when the data size is set to 1.0 with different m values over the two datasets.

In addition to the aforementioned conclusions, varying m values of unseen relations can affect the model’s performance. The experimental data in Table 4 shows that KBPT continually outperformed several baseline methods over two datasets with different numbers of unseen relation labels. In comparison, the proposed model exhibited a more significant superiority when ${\mathrm{m = 10}}$. Such results indicate that the number of unseen relation labels and the scale of the training data had a particular impact on the model’s performance. To further explore the effects of data scale and the relationship between varied m values, different m values were taken to test and observe the changes of ${{\mathrm{F}}_{\mathrm{1}}}$ score. A comparison of the schematic diagrams is shown in Fig. 4. From Fig. 4A, an observation can be made that the improvement of the proposed method became more significant as the data size increased. Fig. 4B indicates that KBPT achieved better performance when m was smaller. In other words, increasing the value of m diminished its advantage. One possible reason for this is that it became more difficult to correctly recognize the relation label with limited knowledge as the number of unseen relation labels increased. In addition, another reasonable inference can be speculated: although KBPT can uncover implicit semantics within each relation, the semantic similarity between relations may lead to confusion in predictions. In other words, the model is more likely to categorize semantically close relations as correct ones. To further demonstrate this supposition, an instance is provided in *Case study*.

As revealed in Fig. 4, the performance in ${{\mathrm{F}}_{\mathrm{1}}}$ score of the KBPT increased gradually with the increase in training data over the two datasets. From Fig. 4A, an observation can be made that the performance increases were more obvious on the Wiki-ZSL dataset. Figure 4B illustrates the change of ${{\mathrm{F}}_{\mathrm{1}}}$ score corresponding to different m values and data sizes on the two datasets. According to Fig. 4B, the proposed model obtained the best performance when m = 10 and the data size was 1.0 for the zero-shot prediction. The specific reasons for this case have been previously explained. In addition, the line chart in Fig. 4B also illustrates the stability and robustness of the proposed model, which consistently outperformed RelationPrompt in ${{\mathrm{F}}_{\mathrm{1}}}$ score across different m values on the two datasets.

**Figure 5:**
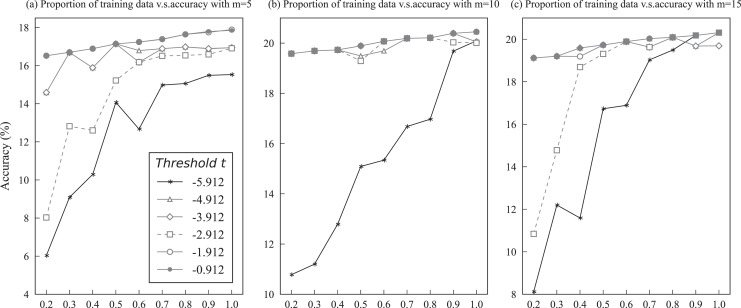
Accuracy of KBPT (our model) with varying threshold value $t$ in the Wiki-ZSL dataset, and (A–C) corresponding to different values of ${\mathrm{m}}$. The x-axis represents the different proportions of training data, and the y-axis represents the percentage of correct trials for tests in each proportion of training data. The best performance is achieved with ${\mathrm{m = 10}}$, in which the training data with different threshold values $t$ achieve a relatively stable and high accuracy.

To further evaluate the effectiveness and stability of KBPT, the hyperparameter sensitivity was also examined. The primary hyper-parameter includes the value of Top-n branches $p$ and threshold $t$ in the multiple triplets decoding. Figure 5 demonstrates the performance of different threshold values $t$ with varying proportions of training data. Notably, only the performance in the accuracy of KBPT on the Wiki-ZSL dataset was reported to evaluate the effect of varying threshold value $t$. This is because the accuracy of other baselines was not suitable for the zero-shot setting, where 100% of the relation labels in the test set are unseen. In Fig. 5, the changes in accuracy values are illustrated by fixing m = {5,10,15} and varying the threshold t and proportion of training data. The scale of training data remained a critical factor affecting the model’s performance, as it determined the quality of parameter updates during the training phase. Moreover, more training data typically led to better performance. From Fig. 5, it can be observed that the model achieved the best performance when ${\mathrm{m = 10}}$, consistent with the findings in Fig. 4B. Additionally, the impact of different $t$ values on the model’s performance was investigated. The experimental results indicate that a minimum value of $t$ was not practical for the model. When $t$ was too small, the search range between positive triplets and negative triplets increased sensitivity. Therefore, during MTD, it was more prone to recognize the wrong relation triplets. Ultimately, $t = - 0.912$ was set to achieve satisfactory results across different datasets with faster search speed.

While the aforementioned experiments effectively verified the impact of varying threshold value $t$ on the performance of the proposed method, the effectiveness of greedily decoding the entire sequence was evaluated by setting different values of branch parameter $p$ in MTD. Specifically, this was done by fixing $m = 10$ and changing the value of branch $p$, so as to obtain the performance changes of the KBPT in accuracy (%) on the Wiki-ZSL dataset. In general, a larger value of $p$ leads to better performance of the model. However, it also results in a slower convergence rate. This is because as the value of $p$ increases, the generation is branched for the head entity or tail entity based on the numbers of p sequences, leading to an exponentially larger search space of $p({\mathrm{triple}}{{\mathrm{t}}_{i,j,z}})$. Consequently, a greedy search becomes inappropriate. Therefore, $p = 4$ was set to achieve satisfactory performance across datasets.

Utilizing single triplet samples in synthetic training data during the triplet extraction phase, the proposed relation extraction model, combined with the MTD approach, effectively tackled the multiple triplet extraction problem by automatically searching and enumerating multiple triplets at inference time. Once again, KBPT outperformed the vanilla prompt tuning model, indicating the critical role of external ontology knowledge as a prompt for generating synthetic training samples. Further, from the experimental data in Table 4, it can be concluded that KBPT on the re-splitter dataset demonstrated effectiveness and robustness for zero-shot relation triplet extraction.

**Figure 6:**
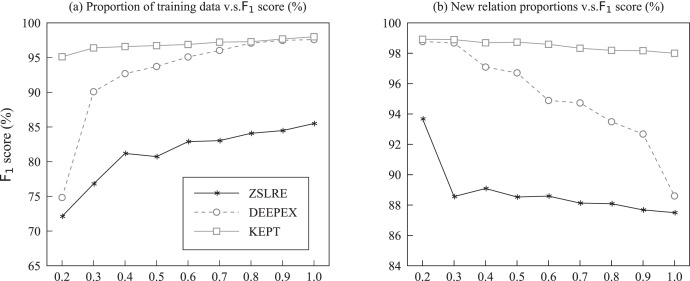
Results of different models with varying proportions of training data (A), and new relation proportions (B) on the NYT dataset. The x-axis indicates the proportion of training data (A) and unseen relation proportions (B), and the y-axis indicates ${{\mathrm{F}}_{\mathrm{1}}}$ score (%). To better highlight the performance of the zero-shot setting, we only select ZSLRE and DEEPEX as the comparative objects.

To evaluate the effectiveness of the KBPT in actual application scenarios, the NYT dataset was re-split and 28 unseen relation labels were set in the test sets. Only the performance of the proposed model was compared with ZSLRE and DEEPEX because other models such as CDNN (Zeng et al., 2014) and REDN do not meet the requirements of the zero-shot setting as outlined in the existing literature. Moreover, the training data and unseen relation label proportions in the test set were changed to observe the varying performance of various models. The broken lines in Fig. 6 demonstrate the performance in ${{\mathrm{F}}_{\mathrm{1}}}$ score of KBPT and other baselines with different variables. From Fig. 6A, an observation can be made that the ${{\mathrm{F}}_{\mathrm{1}}}$ score increased rapidly with the increase of proportions in training data. When increased to a certain extent, the ${{\mathrm{F}}_{\mathrm{1}}}$ score increased slowly and almost remained flat, which is consistent with previous results (Fig. 4A) on Wiki-ZSL and FewRel datasets. From Fig. 6B, an observation can be made that the ${{\mathrm{F}}_{\mathrm{1}}}$ score of all models decreased when the unseen relation proportions increased, and the ${{\mathrm{F}}_{\mathrm{1}}}$ score of DEEPEX dropped most sharply with a drop of 10.17% (98.77–88.60%). However, the performances of KBPT and ZSLRE were relatively stable. The reason is that it is difficult for the model to detect unseen relations. Additionally, unseen relations still existed in the training set during the test phase, and the presence of more new relations in the test set made recognition more challenging. Therefore, the ${{\mathrm{F}}_{\mathrm{1}}}$ score of DEEPEX dropped sharply when the unseen relation proportion reached the requirements of the zero-shot setting. The ${{\mathrm{F}}_{\mathrm{1}}}$ score of the proposed method only decreased around 0.92%, indicating that the proposed method had sufficient robustness when detecting unseen categories. Moreover, KBPT outperformed all of the baselines in ${{\mathrm{F}}_{\mathrm{1}}}$ score and recall rate, demonstrating the achievement of the proposed method.

**Figure 7:**
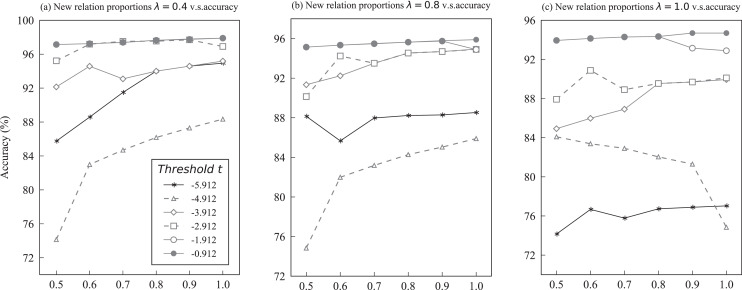
Results of KBPT for varying threshold value $t$ in multiple triplets decoding (MTD) over NYT dataset. This figure shows the accuracy changes by continuously varying the scope of the new relations proportion, *i.e*. $\lambda = \{ 0.4,0.8,1.0\}$. The x-axis indicates the proportion of gradually increasing training data, where we take the minimum proportion as 0.5, and the y-axis represents accuracy (%). The performance of KBPT deteriorates as the proportion of new relation $\lambda$ increases, and the opposite with the proportion of training data, which is consistent with the experimental conclusion in Fig. 6.

In addition to the aforementioned results, the impacts of varying threshold value $t$ on the KBPT were also investigated. The value of threshold $t$ was utilized to filter the final output relation triplets from multiple candidate sequences during the decoding phase. Therefore, different threshold values $t$ from (−5.912, −0.912) were set to test the model’s performance with varying training data on the validation set. Figure 7 shows that the accuracy values were extremely unstable when the threshold value $t$ was between −5.912 and −3.912. By continuously adjusting the range of changes in the threshold value, the final value was set as −0.912. From Fig. 7, an observation can be made that the value of threshold $t$as −0.912, and the performances of KBPT were the most stable. This is likely because when the threshold $t$ was small, the output scores of all candidate triplets would fluctuate significantly, which affected the accuracy of relation prediction during decoding. Additionally, the proportion of new relations $\lambda$ also affected the performance of the proposed model. Further, as the proportion of new relations increased, the performance of the model decreased, consistent with the results of the previous analysis (Fig. 4). Values of new relation proportion $\lambda = \{ 0.4,0.8,1.0\}$ were set, and the threshold value $t$ was fixed as −0.912. The value of accuracy with a maximum difference was 3.2%, corresponding to $\lambda = 0.4$ (97.89%) and $\lambda = 1.0$ (94.69%), respectively.

**Figure 8:**
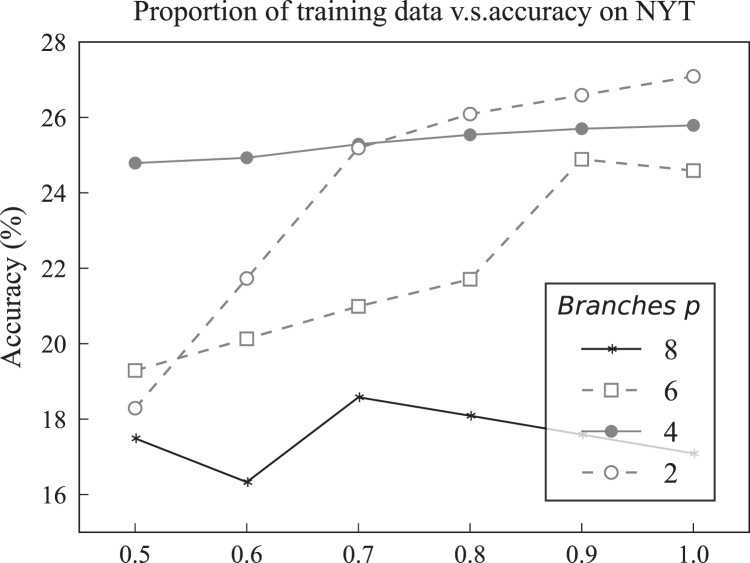
Results for varying branches $p$ with the proportion of training data over the NYT dataset. Setting the value of branch $p$ is 2, 4, 6, 8. Each branch determines the number of generations during the decoding phase. For the convenience of comparison, take the proportions of training data as (0.5, 1.0) (x-axis). The y-axis indicates the accuracy (%). The polylines represent different parameter settings of branch $p$ corresponding to the value of accuracy (%).

To more concretely test the implementation of the proposed method, several accuracy experiments were also performed on the NYT dataset by varying the value of parameter $p$. The plot is shown in Fig. 8. In the test, $p$ indicates the number of branches that can be used to avoid greedy decoding of the entire sequence during the decoding phase, and the computational complexity exponentially increases with the increase in *p* value. That is to say, there will be ${p^3}$ candidate sequences corresponding to each relation triplet. To assess the performance of KBPT, $p = \{ 2,4,6,8\}$ was set. An observation can be made from Fig. 8 that the KBPT reached the most stable status when $p = 4$ and the accuracy performance was higher (25.79%) compared to $p = \{ 6,8\}$. Such results can be attributed to the candidate space of the relation triplets increasing exponentially with the increases in the $p$ value, which resulted in an additional candidate interference of relation triplets. Subsequently, this caused a decline in the accuracy of probability prediction $p({\mathrm{triple}}{{\mathrm{t}}_{i,j,z}})$(that is, the value of accuracy would decrease). On the contrary, according to Fig. 8, the accuracy performances were extremely unstable and fluctuated greatly, although they reached a high accuracy value when $p = 2$. Therefore, we suggest setting the value of $p = 4$ to achieve satisfactory results across datasets.

To provide a more comprehensive evaluation of our model, we also utilize another evaluation strategy to measure the area under the PR curve (AUC) to assess the model’s overall performance. Table 5 reports the AUC performance in open information extraction with supervised methods. Experimental results indicate that our model outperforms the fully supervised methods and zero-shot setting model in terms of ${{\mathrm{F}}_{\mathrm{1}}}$ score and AUC. The average AUC value can be calculated as follows according to the literature (Cortes & Mohri, 2003).

**Table 5:** Results of different models with supervised learning and the zero-shot setting on NYT dataset (%). The asterisk (*) represents the use of a supervised method. The best result is highlighted in boldface.

Model	${{\mathrm{F}}_{\mathrm{1}}}$(%)	Accuracy(%)	AUC(%)
MAMA**	32.90	94.73	9.40
DEEPEX	85.50	95.60	72.50
KBPT (our model)	**98.00**	**97.54**	**81.35**

Table 5 lists the experimental results of the different models with supervised learning and zero-shot setting on the NYT dataset. From Table 5, it can be found that the proposed model achieved state-of-the-art performance on the NYT dataset and also outperformed the supervised learning method by 2.81% in accuracy and 71.95% in AUC.

The following conclusions can be drawn from the experimental data in Table 6. First, the KBPT in the zero-shot setting outperformed the supervised learning scenarios in k = 16 on the validation and test sets. Moreover, the proposed model yielded 6.88% and 9.06% absolute improvements compared with AdaPrompt-tuning on the full dataset, being less than the zero-shot setting but still considerable. Second, KBPT significantly outperformed the SOTA model in the application scenario of a few-shot setting, both k = 8 and k = 16. Additionally, the proposed model achieved a definite improvement of 2.35% (and 1.495% on average) compared to the current SOTA model, AdaPrompt-tuning, in the setting of k = 16. Thirdly, KBPT exhibited robustness comparable to the current SOTA model. In summary, KBPT achieved state-of-the-art performance in various settings, and this can be attributed to the ontology knowledge and relation labels injected into the prompt template, which contain rich semantic information.

**Table 6:** Results on TACRED-Revisit dataset with few-shot and zero-shot setting ( ${{\mathrm{F}}_{\mathrm{1}}}$ score %). $k = \{ 8,16,32\}$ represents the number of instances per class in the few-shot setting. k = 0 represents the zero-shot setting. Full denotes the complete training set used. The results for k = 0 are our re-implementation experiments on the TACRED-Revisit dataset, and the results of $k = \{ 8,16,32\}$ are directly copied from the original published literature. The best result is highlighted in boldface.

Model	k = 0		k = 8		k = 16		k = 32		Full	
	Vali	Test	Vali	Test	Vali	Test	Vali	Test	Vali	Test
SpanBERT	8.0	6.3	9.4	7.2	18.3	16.2	29.8	25.8	–	78.0
GDPNet	7.85	5.76	9.1	7.3	19.3	17.8	30.2	26.1	–	80.2
AdaPrompt-tuning	24.34	22.05	26.6	25.20	29.5	27.3	32.9	30.8	81.3	80.8
KBPT (our model)	**31.14**	**29.95**	**35.34**	**30.13**	**36.73**	**32.56**	**40.15**	**38.84**	**88.18**	**89.86**

**Figure 9:**
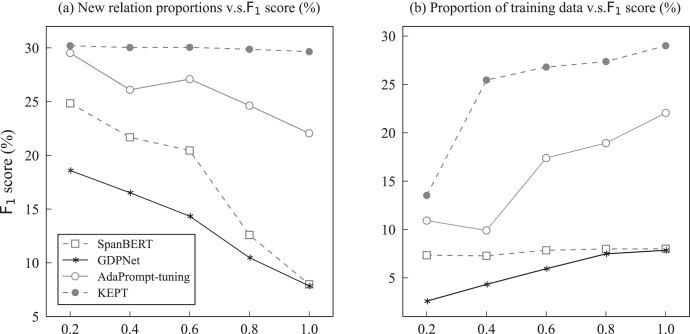
Comparison between the supervised learning and our proposed model when varying new relation proportions (A) and the proportion of training data (B). The x-axis indicates the proportions of the new relation (A) and the proportion of training data (B), that is, the zero-shot setting of the model when the proportion of the new relation is 1.0. The y-axis indicates the ${{\mathrm{F}}_{\mathrm{1}}}$ score (%). For the continuously increasing proportion of new relations, the performance of the KBPT is relatively stable, reflecting the advantages of our proposed model for the zero-shot setting.

In addition to the aforementioned experimental results, it was also examined how varying proportions of training samples and new relations affect the model’s performance. Fig. 9 shows the ${{\mathrm{F}}_{\mathrm{1}}}$ score trend of several models in the test dataset with various fractions of training samples and new relations. From Fig. 9A, an observation can be made that the performance of KBPT was relatively stable for the continuously increasing proportion of new relations, reflecting the advantages of the proposed model for the zero-shot setting. In contrast, other supervised learning models were susceptible to increased relation proportion. That is to say, as the proportion of new relations continued to increase, the performance of these models dropped sharply. Moreover, the performances of different models were compared with the varying proportions of training samples. Figure 9B shows that the KBPT outperformed all the supervised learning methods in various proportions of training samples. In addition, the ${{\mathrm{F}}_{\mathrm{1}}}$ score growth rate was the fastest compared with other models, resulting in a faster convergence during the training phase. As such, the described experimental results indicate the effectiveness and sustainability of the proposed model for zero-shot settings. In summary, the adaptability of the zero-shot setting to unknown relation types was verified. Exploring methods to recognize additional new relations in real-world scenarios is an area worth investigating in future work.

### Ablation studies

To further highlight the roles of different components in KBPT, we perform the ablation experiments on the validation set of Wiki-ZSL and FewRel with 10 unknown relations ( $m = 10$), and the experimental results are given in Table 7. Specifically, we examine the performance of external ontology knowledge (EOK), SAAN, virtual tokens (VT), multiple triplets decoding (MTD), and fine-tuning on synthetic samples (FTSS).

**Table 7:** Results of ablation studies over different components of KBPT on Wiki-ZSL and FewRel validation sets. w/o EOK indicates the model without External Ontology Knowledge, and so on down the line. A minus sign in parentheses indicates the drop percentage. The best result is highlighted in boldface.

Model	Wiki-ZSL			FewRel		
	Pre(%)	Rec(%)	${{\mathrm{F}}_{\mathrm{1}}}$(%)	Pre(%)	Rec(%)	${{\mathrm{F}}_{\mathrm{1}}}$(%)
Full Method (KBPT)	34.56	40.86	**37.45**	27.43	31.61	**29.37**
w/o SAAN	33.28	39.37	36.07 (−1.38)	26.57	30.31	28.32 (−1.05)
w/o FTSS	31.67	39.71	35.24 (−2.21)	26.13	28.93	27.46 (−1.91)
w/o EOK	25.48	31.77	**28.28 (−9.17)**	20.53	21.85	**21.17 (−8.20)**
w/o MTD	28.46	33.24	30.67 (−6.78)	23.19	23.71	23.45 (−5.92)
w/o VT	30.52	36.42	33.21 (−4.24)	25.89	27.37	26.61 (−2.76)

**Effect of external ontology knowledge (EOK):** To demonstrate the effects of external ontology knowledge on prompt tuning, ablation experiments were conducted involving the Wiki-ZSL and FewRel validation sets, and the results are shown in Table 7. Specifically, when only the relation label and virtual tokens were employed as prompts, and external ontological knowledge was removed, the ${{\mathrm{F}}_{\mathrm{1}}}$ score dropped from 37.45% to 28.28% on Wiki-ZSL and from 29.37% to 21.17% on FewRel (the bold part in Table 7), respectively. Although relation labels contain rich semantic information that can prompt language models to generate synthetic training instances, injecting ontology knowledge as additional prompts can further enhance the discovery of relation-related knowledge, which had the most significant effect on the entire experiment. The experimental results reveal that injecting ontological knowledge to express the desired relations was critical for generating synthetic training instances in zero-shot scenarios.

**Effect of multiple triplet decoding (MTD):** An ablation study was also conducted to validate the effectiveness of the decoding method on both of the validation sets. Specifically:

- Traversing all tokens in the sentence and recognizing all entity categories and relation labels to generate the triplet sequence. Instances are discarded if part of the generated sequence has an invalid format.
- Utilizing the Greedy matching algorithm to dynamically match the input sentence and the output sequence at the token level, and then using the Needleman-Wunsch alignment algorithm to recognize the token span corresponding to entities in the original sentence.
- Iterating through all words to seek the semantically closest token span to match the predicted tail entity for each relation in the output sentence. The relation label is discarded if such a token span does not exist. Finally, the extracted triplets, including relations and entity pairs, are discarded if they are not contained in the original dataset.

The relatively large performance gap indicates that MTD is vital for multiple triplet extraction in zero-shot settings. Moreover, the complete model outperformed the w/o MTD by 6.78% and 5.92% in ${{\mathrm{F}}_{\mathrm{1}}}$ score on Wiki-ZSL and FewRel, respectively. The results suggest that the hierarchy and enumeration of multiple candidate schemes are of comparatively high quality and demonstrate MTD’s applicability to extract multiple relation triplets.

**Effect of virtual tokens (VT):** Ablation experiments were also performed to validate the effectiveness of virtual tokens on both sides of the relation label. Specifically, the relation label and external ontology knowledge were reserved in the prompt template, removing the specific virtual tokens and randomly initializing the position of the original virtual tokens. From the experimental data in Table 7, an observation can be made that removing the virtual tokens seriously affected the model’s performance, causing the ${{\mathrm{F}}_{\mathrm{1}}}$ score to decrease by 4.24% on Wiki-ZSL and by 2.76% on FewRel, respectively. Moreover, such results also suggest that preserving semantic information in relation labels as prompt embedding is crucial for extracting multiple relation triplets, especially in low-resource regimes.

**Figure 10:**
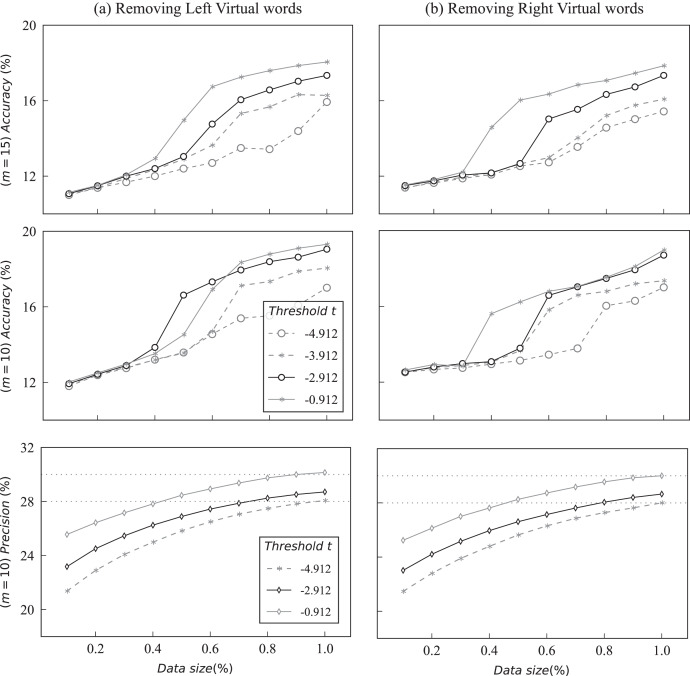
Precision and accuracy (y-axes) of KBPT with varying proportions of training data (x-axes) on Wiki-ZSL. The first three illustrations on the left represent the performance (m = 10, 15) of removing the left virtual tokens in the prompt template, and the right represents the performance of removing the virtual tokens on the right. Each line indicates performance for a specific threshold value $t$. To measure the accuracy (%), we take four specific threshold values for comparison when m = 10 and m = 15, respectively. To measure the precision (%), we only take three specific thresholds when m = 10. Compared with the complete KBPT, the performances of the model without virtual tokens are decreased as a whole.

Previous studies (Chen et al., 2022a; Chia et al., 2022) have demonstrated that relation labels contain rich semantic knowledge. To effectively integrate this semantic knowledge into the prompt template, we conducted a series of operations. First, a vocabulary of virtual tokens was constructed based on the relation labels, with the specific format of virtual tokens shown in Table 8. Each relation label contained important entity information such as person, location, time, organization, and others. Thus, the head layer of PLMs was extended with learning relation representations to generate the virtual tokens. Second, to assess the effectiveness of the injected virtual tokens, several comparative experiments were conducted on Wiki-ZSL, and the performances are illustrated in Fig. 10. Removing the virtual tokens on the left of the relation label, the performances of the model are shown in Fig. 10A. An observation can be made that the performances of the model declined after removing the virtual tokens by comparing them with the complete model. In particular, the accuracy value dropped by 2.26% ( $m = 15$), and precision dropped by 2.32% when the threshold $t = - 0.912$. From Fig. 10B, it is evident that the performance of the proposed model deteriorated, decreasing to 18.97% when $m = 10$ in accuracy. It is speculated that this outcome is related to the position of the relation label, and the longer the sentence, the richer the relational semantics represented. Overall, the experimental results illustrate the effectiveness of our designed virtual tokens for knowledge injection in extracting multiple relation triplets.

**Table 8:** Instances of relation labels in the TACRED-Revisit dataset. ${W_{sub}}$ and ${W_{obj}}$ indicate the subject and object in relation triplets, respectively, and ${W_v}$ indicates the reconstructed relation tokens used for the virtual tokens.

Relation labels	${W_{sub}}$	${W_{obj}}$	${W_v}$ (Disassembled Relation Prepared for Virtual Words)
org: country_of_birth	Person	Country	{“country”, “of”, “birth”}
org: city_of_death	Person	City	{“city”, “of”, “death”}
org: country_of_citizenship	City	Country	{“country”, “of”, “citizenship”}
org: countries_of_residence	Person	Country	{“countries”, “of”, “residence”}
org: country_of_headquarters	Country	City	{“country”, “of”, “headquarters”}
org: top_members/employees	Department	Person	{“top”, “members”, “employees”}
org: city_of_headquarters	City	Region	{“city”, “of”, “headquarters”}
org: stateorprovince_of_headquarters	State	City	{“stateorprovince”, “of”, “headquarters”}
per: stateorprovince_of_birth	Person	City	{“stateorprovince”, “of”, “birth”}
per: cause_of_death	Person	Event	{“cause”, “of”, “death”}

**Effect of fine-tuning on synthetic samples (FTSS):** Under normal circumstances, fine-tuning was initially conducted on seen relation instances from the training set, followed by final tuning on generated synthetic samples for the unseen relation. Parameters were only fine-tuned on seen relation instances from the training set to validate the effectiveness of fine-tuning. Table 7 reveals a notable drop in ${{\mathrm{F}}_{\mathrm{1}}}$ score on both datasets, significantly impacting performance without fine-tuning on unseen relation samples. These results suggest that final fine-tuning for unseen relations played a supporting role in identifying the correct relation category in zero-shot setting scenarios. Optimal results were achieved by separately fine-tuning the seen and unseen relations.

**Effect of fine-tuning on SAAN:** SAAN was added between the $L - 1$ layer and *L* layer of the PLM to improve the semantic interaction and fusion of the learning representations. To facilitate verification and comparison of the results, only this module was removed at the triplet extraction stage. Specifically, the input prompt embedding and text representations were fed into the L layer of PLM to fuse these representations and then input them into the decoding layer. The experimental data are reported in Table 7. An observation can be made that removing the SAAN resulted in a considerable performance drop, as the syntax-aware attention mechanism could utilize the fused knowledge representations from input text to aggregate more effective external knowledge.

**Figure 11:**
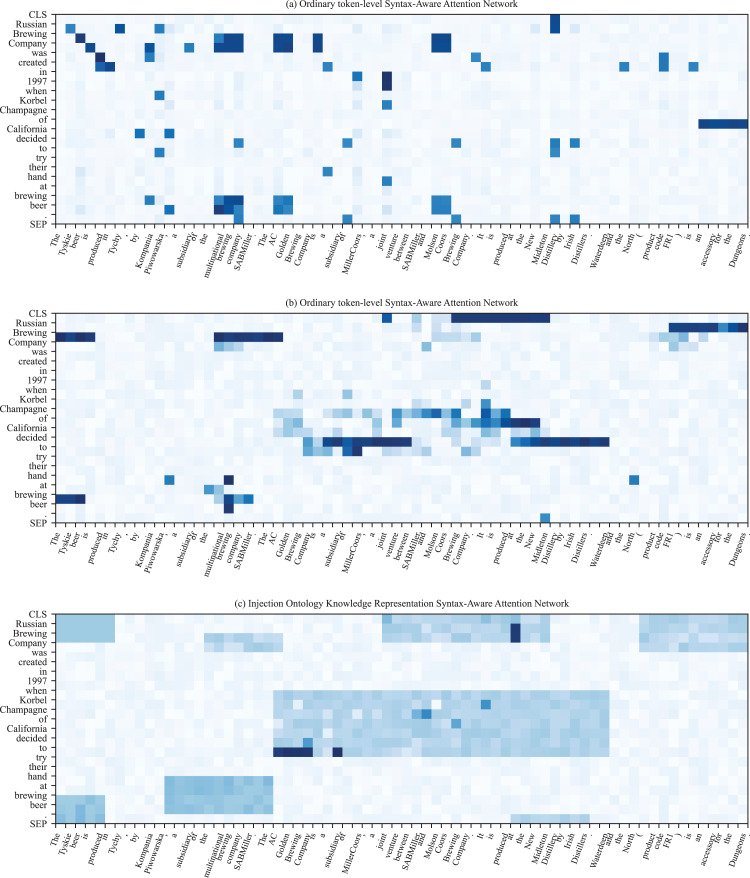
Instance attention visualizations for different components of SAAN training with BERT PLM on Wiki-ZSL at relation triplet extraction phase. (A) Indicates ordinary token-level attention network, (B) indicates ordinary token-level SAAN, and (C) indicates injected ontology knowledge representations SAAN(our model). The attention distributions are taken from the layer of Triplet decoding and maxed over the representation matrix. The attention distribution in (A) is quite scattered and cannot focus on enough of the context information related to the token *created*. The attention distribution in (B) slightly alleviates the problem in (a), but the attention distribution is uneven to focus on the context information of tokens *Brewing Company*. The SAAN that injected ontology knowledge representations (our model) solves this problem well.

**Visualization of different attention networks with BERT:** To further verify the effectiveness of the SAAN, the attention weight matrices learned in a sentence were visualized to illustrate the capability of syntactic context representations. In Fig. 11, the association of the relation in a sentence trained on BERT PLM is visualized. A comparison was made among the visualized attention weight matrices generated by the ordinary token-level Attention Network in Fig. 11A, the ordinary token-level SAAN in Fig. 11B, and the injected ontology knowledge representations SAAN (the proposed model) in Fig. 11C, respectively. In Fig. 11A, the learned token representations of the ordinary attention mechanism appear quite scattered and do not clearly represent relational semantics. For instance, the model directed attention towards more tokens, such as the verb *(was, is)* or the preposition *of*, which are not highly relevant to the relation label Created in. Additionally, a single token attention representation lacks more semantic information. The ordinary token-level SAAN in Fig. 11B slightly alleviated this problem because it included semantic similarity matrices that can focus on more syntactic context. The proposed model (Fig. 11C) can focus on contextual, semantic, and syntactic information related to the relation *Created in* such as *Brewing Company*. Further, the injection of ontology knowledge representation in the SAAN also contributes to aggregating relations as ensemble information.

**Figure 12:**
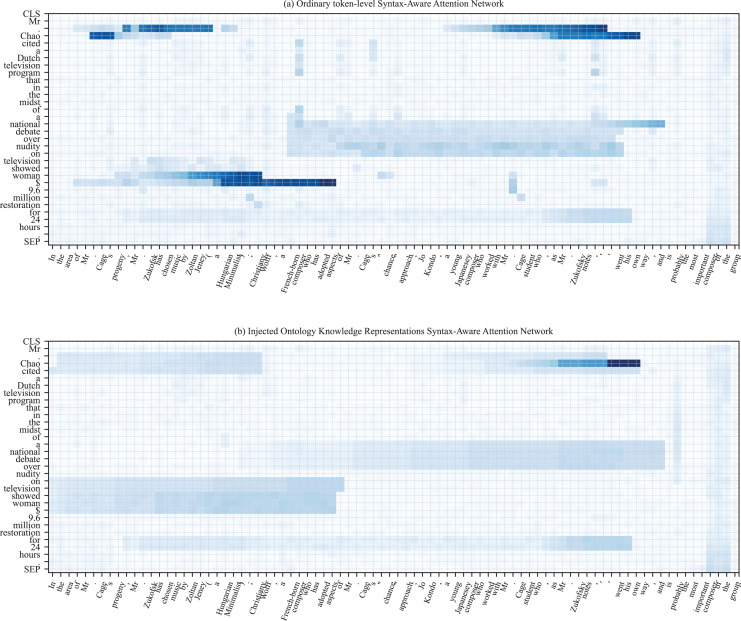
Instance attention visualizations of attention weight matrices for different components of the SAAN with GPT-2 PLM are training on Wiki-ZSL. (A) Indicates ordinary token-level SAAN, (B) indicates the injection ontology knowledge representations SAAN (our model). The attention distributions are also taken from the layer of triplet decoding and maxed over the representation matrix. The attention distribution in (A) is scattered and cannot focus enough on the entity representations of syntactic contexts, such as the character entities (*e.g*., Mr.Chao). The SAAN that injected ontology knowledge representations (our model) in (B) solves this problem well.

**Visualization of different attention networks with GPT-2:** Due to the focus of the present study being related to the PLM and prompt tuning, the SAAN was extended to other autoregressive language models (LMs) such as GPT-2. Specifically, external ontological knowledge and relation label embedding were injected into the input sequence for GPT-2, and the relation label in PLMs was further utilized to generate the virtual tokens. A series of experiments were performed on the Wiki-ZSL dataset, and the visualized renderings of weight matrixes in different sentences are shown in Fig. 12. For the convenience of comparison, the ordinary token-level SAAN is presented in Fig. 12A and the injected ontology knowledge representations SAAN (the proposed model) are shown in Fig. 12B. In Fig. 12A, it can be observed that the model can focus well on entities related to the relation characters, such as *Mr.Zukofsk* and *television program* due to the semantic similarity matrix in SAAN. Additionally, it incorporates the most relevant relation semantic crucial for multiple relation triplet extraction. Injecting ontology knowledge with the SAAN in Fig. 12B effectively aggregated semantic information related to relations and entities. Notably, the effectiveness is comparable to training with BERT on the Wiki-ZSL dataset. These results also indicate that the proposed methods are model-agnostic and can be applied across different kinds of PLMs.

### Case study

To further verify the effectiveness of generated synthetic data in the zero-shot setting, the investigation involved varying numbers of generated synthetic training samples. The variable parameters were evaluated on the Wiki-ZSL validation set, which includes 10 unseen relations. Conclusions were drawn based on the results obtained. First, the model performance was affected by the quality of the synthetic samples, and with the increase in the synthetic samples’ amount, the model performance was improved. However, the performance no longer improved when the number of synthetic samples increased to a certain extent. In addition, the experimental results show that when the number of synthetic samples corresponding to each relation label increased from 150 to 300, the model’s performance ( ${{\mathrm{F}}_{\mathrm{1}}}$ score) continued to grow. Instead of increasing, the performance decreased when the number of synthetic samples increased from 300 to 3,000. Consequently, we decided to set the final number of synthetic samples to 300. These results suggest that while having more synthetic samples can benefit the extraction of multiple relation triplets, an excessive number of training samples can also introduce data noise, leading to a decline in performance. Second, the diversity of synthetic training samples was also investigated. This involved assessing the number of unique entities and words present in synthetic and real instances on the Wiki-ZSL validation set as the evaluation metric. Specifically, 10 relation labels were selected to generate an equal number of synthetic samples, and then a comparison was made. The experimental results reveal that the generated synthetic samples exhibited lower diversity in unique words but richer diversity in unique entities compared to real instances. This can be attributed to the uniqueness of entity names, which the model pays attention to extensively during pre-training. Additionally, the use of relation prompts aids the model in generating training instances for unseen relation labels, thereby constraining the diversity of contextual information in the synthetic samples. The real instances and generated synthetic samples on the Wiki-ZSL dataset are presented in Table 9.

**Table 9:** Case study of relation labels between real instances and generated synthetic instances on Wiki-ZSL dataset. The head and tail entries are shown in bold. The // is used to split the two generated synthetic samples.

Relation labels	Real instances	Generated synthetic samples
Founded by	**Kamuzu Academy** is a private boarding school in Malawi that was founded by, and named after, the late **Hastings Kamuzu Banda**, the former President of Malawi.	**MicroProse** was an American video game publisher and developer founded by Wild Bill Stealey and **Sid Meier** in 1982 as MicroProse Software Inc. // **Samuel de Champlain** founded **Quebec** (1608) and explored the Great Lakes.
Location of birth	**Lurleen Brigham Burns** Wallace (September 19, 1926 May 7, 1968), born in **Tuscaloosa, Alabama**, was the 46th governor of Alabama from 1967 until her death in 1968.	**Nova Scotia** was also the birthplace and home of **Samuel Cunard**, a British shipping magnate, born at Halifax.// Later his family moved to **Sheikhupura** (now in Pakistan), close to Nankana Sahib, the birthplace of the founder of **Sikhism** (Guru Nanak).
Country of citizenship	**He** is from a Greek shipping family, grew up in London and has **British** citizenship, and has lived in New York City since 2004.	**Joseph Francis Busch** (April 18, 1866 & mdash; May 31, 1953) was an **American** prelate of the Catholic Church.// His work displayed his support for the Conservative Party of **Canada** and criticized Liberals such as **Wilfrid Laurier**, as well as French Canadians, Catholics, and Americans.
Member of sports team	Hu00e5kon **Skogseid** (born 14 January 1988 in Bu00e6rum) is a Norwegian football defender are playing for **Lillestr** u00f8m.	Hu00e5kon While in Spain, **Billy** idolised **England national team** star Wayne Rooney and Spanish forward Fernando Torres to whom he compared himself to.// Owen played five seasons with **the Bruins**, pairing on defense with players including Lionel Hitchman and **Eddie Shore**, and won the Stanley Cup with the team in 1929.

In addition to the described case study of generated synthetic instances, a series of experiments were conducted and the process of multiple triplet extraction when m = 10 was reported. The experimental results are shown in Table 10 to illustrate the significant role of different components in the proposed model. From Table 10, several findings can be observed. First, removing external knowledge prompts hurt the performance heavily. Specifically, the model could not identify the relation *member of the sports team* and the extracted entities, and the recognized relations did not correspond. Further, this observation may be attributed to the quality of the generated synthetic instances. Without the prompt of external ontology knowledge, the synthetic samples experience a decline in quality due to noise. Given the abundance of generated synthetic samples, they are not listed in Table 10. This finding also highlights the benefit of injecting external ontology knowledge for enhancing relation representation. Second, the injection of virtual tokens plays a more significant role compared to the relation label prompt as the ${{\mathrm{F}}_{\mathrm{1}}}$ score differed by 10% between them. This also indicates that there was an amount of relation semantic information contained in virtual tokens. Finally, removing both the ontology knowledge and virtual tokens caused a severe performance decrease (−6.50%). Further, the model did not identify the main relation triplet {relation: member of sports team, Head entity: She, Tail entity: national championship team}, and extracted the wrong relation type {relation: attendance, Head entity: Sherri Turner, Tail entity: LPG}. These results reveal virtual tokens play a positive role in prompting the relation semantic representation.

**Table 10:** Case study results on Wiki-ZSL dataset. The Input Instances include input original and prompt text. Relation labels and virtual words are bracketed in underlined and italics, respectively. The symbol w/o indicates removing the corresponding component in the model. The head and tail entities are shown in bold. The relation label is shown in italics in the Relation Triplets column.

Model	Input instances	Relation triplets	${{\mathrm{F}}_{\mathrm{1}}}$ score(%)
KBPT (w/o Ontology Knowledge Prompt)	[CLS] She played her collegiate golf at Furman University, and was on the 1976 national championship team that included future LPGA players Betsy King, Sherri Turner and Cindy Ferro.[SEP] [member of] *[member of sport team]* [sport team] [SEP]	Head Entity: **Cindy Ferro**/**Sherri Turner**/**She** Tail Entity: **Furman University**/**1976**/**LPGA** Relation: *country of citizenship*/*date of* birth/*attendance*	27.17
KBPT (w/o Relation Label Prompt)	[CLS] She played her collegiate golf at Furman University, and was on the 1976 national championship team that included future LPGA players Betsy King, Sherri Turner and Cindy Ferro.[SEP] [member of] [sport team] [SEP] sports teams or clubs that the subject currently represents or formerly represented…….[SEP]	Head Entity: **She**/**Betsy King** Tail Entity: **national championship team**/**Furman University** Relation: *member of sports team*/*educated at*	28.34
KBPT (w/o Virtual Words Prompt)	[CLS] She played her collegiate golf at Furman University, and was on the 1976 national championship team that included future LPGA players Betsy King, Sherri Turner and Cindy Ferro.[SEP] *[member of sport team]* [SEP] sports teams or clubs that the subject currently represents or formerly represented…..[SEP]	Head Entity: **She**/**Betsy King** Tail Entity: **national championship team**/**Furman University**/**LPGA** Relation: *member of sports team*/*member of sports team*	28.09
KBPT (w/o Ontology Knowledge and Virtual Words Prompt)	[CLS] She played her collegiate golf at Furman University, and was on the 1976 national championship team that included future LPGA players Betsy King, Sherri Turner and Cindy Ferro.[SEP] *[member of sport team]* [SEP]	Head Entity: **She**/**Sherri Turner** Tail Entity: **Furman**/**LPGA** Relation: *country of citizenship*/*attendance*	26.67

### Discussion and analysis

**Qualitative analysis of the generated synthetic samples:** Table 9 lists several instances of real samples and generated synthetic samples to evaluate how to generalize entity instances in the ground from the relation labels. The relation labels and the real instances were taken from the factual materials in the Wiki-ZSL dataset. For simplicity, only four relation labels are presented, and each of them corresponds to two randomly generated synthetic samples that were segmented with //. In most instances, the model was able to understand the correct semantics of relation labels and generate synthetic samples similar to real instances. The analysis of the four relation labels *founded by*, *location of birth*, *country of citizenship*, and *member of sports team* in Table 9 reveals notable findings. For the first three relation labels, the entity pairs in the generated training samples accurately corresponded to the relation labels, and the sentences’ meaning aligned with semantic logic. However, for the last relation label, *member of sports team*, the generated entity pairs failed to form the correct relation triplet with the given relation label, despite being related to sports text. In other words, the generated text represented a relation label related to being a *member of sports team*. This study’s outcome highlights that there is ample room for improvement in the quality of generated synthetic samples. Future research should focus on generating appropriate head and tail entities to achieve more precise matching.

**Can KBPT apply to other LMs?:** Since the primary research goal of the present study was prompt-tuning for PLMs, the proposed method was extended to autoregressive language models such as RoBERTa and GPT-2. To achieve this, the prompt template and external ontology knowledge were directly appended to the input text. Subsequently, this combined input was fed into the PLMs for encoding to generate the required synthetic samples through the softmax layer during the generation stage. Subsequently, the same PLM for fine-tuning was utilized to generate relation triplets during the extraction phase. Notably, the new parameters were introduced in the model’s head during the generation and extraction stage. Therefore, RoBERT and GPT-2 were able to generate relatively high-quality synthetic samples with the assistance of external knowledge and prompt templates. However, fine-tuning tends to result in poor performance in the low data regime of triplet extraction. In contrast, KBPT based on RoBERTa and GPT-2 can overcome this challenge and achieve significant improvement. As shown in Fig. 12, the visualization of the SAAN with GPT-2 can focus on more relational semantic information from the context. This suggests that the proposed method is not only model-agnostic but also can fully exploit the text representation ability in the PLM.

**Interpreting representation space of virtual tokens:** Further research is needed to explore the optimized representation space of virtual tokens because these virtual tokens are initialized with semantic information in the relation labels. The specific approach involves firstly initializing the virtual tokens with the average value of entity pairs ( $\{ {e_h},{e_t}\}$) embedding in the candidate set and then projecting them into the vocabulary space of the PLM, which can fully utilize the semantic knowledge contained in the relation labels to assist the model in generating high-quality synthetic samples. To this end, a series of experiments were conducted to analyze the specific semantic representations of selected virtual tokens. Additionally, the relationship between virtual tokens and entity categories was verified to clarify how these tokens can adaptively correspond to the entity categories sourced from the context, as shown in Table 8. Concretely, the head of the Transformer in PLMs was utilized to generate virtual tokens and select the top-3 tokens according to the ${L_2}$ distance embedding based on vocabulary space. From these experiments, due to synergistic optimization with knowledge representation limitations, these learned virtual words could be dynamically adjusted with the context and play a supervised role in the relation triplet extraction. This phenomenon also provided inspiration to further expand the representation learning of prototype relations into the prompt template in future studies. The designed prompt can be applied to other NLP tasks such as event extraction and knowledge graph completion.

## Conclusion and future work

In the present study, a novel method called Knowledge-Based Prompt Tuning (KBPT) was proposed for zero-shot relation triplet extraction, which breaks through the bottleneck of previous task settings and encourages further investigation in low-resource regimes. The proposed model effectively addresses the zero-shot setting problem in multiple relation triplet extraction. First, to address the incompleteness of prior knowledge, ontology knowledge and relation labels were incorporated into the prompt template to enrich relation semantics for generating synthetic samples. Secondly, the embedding representations were synergistically optimized through collective training to alleviate the knowledge heterogeneity issue. Thirdly, multiple triplets decoding (MTD) was proposed as a solution to the challenge of extracting multiple relation triplets in a sentence. Finally, experimental results indicate that KBPT significantly outperformed prior zero-shot relation triplet extraction methods on four publicly available datasets, setting the baseline for future work.

As mentioned in Discussion and analysis, there is ample room for further research in improving the quality of generated synthetic data. Future endeavors could focus on enhancing the precision of matching between the generated entity pairs and relation labels. This refinement could significantly enhance the applicability of the generated data for various zero-shot information extraction tasks.

## Supplemental Information

10.7717/peerj-cs.2014/supp-1 Supplemental Information 1 Script files.

10.7717/peerj-cs.2014/supp-2 Supplemental Information 2 Encoder.

10.7717/peerj-cs.2014/supp-3 Supplemental Information 3 Wrapper.

10.7717/peerj-cs.2014/supp-4 Supplemental Information 4 Generation function.

10.7717/peerj-cs.2014/supp-5 Supplemental Information 5 Model.

10.7717/peerj-cs.2014/supp-6 Supplemental Information 6 Label type.

10.7717/peerj-cs.2014/supp-7 Supplemental Information 7 Test set.

## Additional Information and Declarations

### Data Availability

The following information was supplied regarding data availability: FewRel is available at https://thunlp.GitHub.io/1/fewrel1.html NYT is available at https://GitHub.com/davidsbatista/Annotated-Semantic-Relationships-Datasets/blob/master/datasets/DataSet-IJCNLP2011.tar.gz TACRED-Revisit is available at https://paperswithcode.com/dataset/tacred-revisited Wiki-ZSL is available at https://raw.GitHubusercontent.com/dinobby/ZS-BERT/master/resources/property_list.html The code is available at GitHub and Zenodo: - https://GitHub.com/Phevos75/KBPT - Guo Qian. (2024). Phevos75/KBPT: Second release of my KBPT (v1.0.1). Zenodo. https://doi.org/10.5281/zenodo.10783953.
